# A bibliometric analysis of neuroimaging studies on cognitive control in autism spectrum disorder (2000–2025)

**DOI:** 10.3389/fpsyt.2026.1765161

**Published:** 2026-05-26

**Authors:** Jing Hu, Jiawei Zhao, Hao-Jie Chen, Xinyue Zhang, Junhua Dang

**Affiliations:** 1 Faculty of Psychology, Tianjin Normal University, Tianjin, China; 2 Institute of Social Psychology, School of Humanities and Social Sciences, Xi’an Jiaotong University, Xi’an, China; 3 State Key Laboratory of Cognitive Neuroscience and Learning and IDG/McGovern Institute for Brain Research, Beijing Normal University, Beijing, China; 4 Division of Life Science and State Key Laboratory of Nervous System Disorders, The Hong Kong University of Science and Technology, Hong Kong, Hong Kong SAR, China; 5 Department of Surgical Sciences, Uppsala University, Uppsala, Sweden

**Keywords:** autism spectrum disorder, bibliometrics, cognitive control, functional connectivity, neuroimaging, transdiagnostic, triple network model

## Abstract

**Objective:**

This study aims to systematically analyze neuroimaging research on cognitive control in Autism spectrum disorder (ASD) from 2000 to 2025 using bibliometric methods, in order to reveal the evolutionary trajectory, core knowledge base, research hotspots, and future frontiers of the field.

**Methods:**

A search was conducted on the Web of Science Core Collection and Scopus databases, resulting in the inclusion of 1,581 relevant articles. VOSviewer and the Bibliometrix package in R were utilized to conduct a comprehensive visualization and quantitative analysis of annual publication volume, country/institution/author collaboration networks, keyword co-occurrence, document co-citation, and thematic evolution.

**Results:**

(1) The volume of research literature showed exponential growth, with an annual growth rate of 21.61%, entering a period of rapid development particularly after 2012, which is closely related to the popularization of functional magnetic resonance imaging (fMRI) technology. (2) “Functional connectivity,” “executive function,” and “default mode network” were the most central keywords. “Functional connectivity” rapidly became a hub connecting various themes after 2010, marking a paradigm shift from “functional localization” to “brain network dysregulation.” (3) The “Triple network model” proposed by Menon was the most cited document, laying the core theoretical foundation for understanding ASD as a disorder of large-scale brain network dysfunction. (4) “Transdiagnostic” research has emerged as a new hotspot, while “multimodal imaging,” “machine learning,” and “dynamic connectivity” represent highly promising future directions.

**Conclusion:**

Over the past two decades, neuroimaging research on cognitive control in ASD has undergone a profound paradigm shift: from focusing on abnormal activation in isolated brain regions to exploring the static and dynamic dysregulation of large-scale brain networks. The research perspective has also expanded from a single-disorder model to a transdiagnostic framework that includes comparisons with other neurodevelopmental disorders (e.g., ADHD). Future research should focus on the fusion of multimodal data, the application of computational psychiatry methods, and the translation of basic research findings into personalized clinical interventions.

## 1 Introduction

Autism spectrum disorder (ASD) is a complex neurodevelopmental disorder characterized by core clinical features of deficits in social communication and interaction, as well as restricted, repetitive patterns of behavior, interests, or activities (1). In recent years, the global prevalence of ASD has shown a significant upward trend, becoming a serious public health challenge. According to a 2023 report from the U.S. Centers for Disease Control and Prevention based on 2020 data, the prevalence of ASD among 8-year-old children in the United States reached as high as 1 in 36, a stark increase compared to the 1 in 150 ratio in 2000 (2). This growing trend not only reflects increased public awareness and refined diagnostic criteria but also underscores the urgent need to investigate its pathophysiological mechanisms (3).

Cognitive control, a core component of executive function, encompasses a series of higher-order cognitive processes such as working memory, inhibitory control, and cognitive flexibility, which are fundamental for individuals to regulate thoughts and control behaviors in adapting to complex and changing environments (4, 5). Substantial evidence indicates that cognitive control deficits are a common feature in individuals with ASD and are closely related to their social difficulties and repetitive behaviors (6–9). For instance, insufficient cognitive flexibility may lead to difficulties in adapting to environmental changes or shifting mindsets, while deficits in inhibitory control may be associated with the persistence of repetitive behaviors (4).

Neuroimaging techniques, particularly functional magnetic resonance imaging (fMRI) and diffusion tensor imaging (DTI), have opened new avenues for non-invasively studying the neural mechanisms of cognitive control in ASD (10). Through these technologies, researchers can examine the brain activity patterns, structural connectivity, and functional connectivity features of individuals with ASD during cognitive tasks or in a resting state (8, 11, 12). While early studies often focused on abnormal activation in specific brain regions, the research focus in recent years has gradually shifted towards the dysfunction of large-scale brain networks. For example, compared to typically developing children, children with ASD show decreased activation in the right middle temporal gyrus but increased activation in the mid-dorsal cingulate cortex/superior frontal gyrus, left middle frontal gyrus, and right inferior frontal gyrus during tasks related to cognitive control (8). This suggests that although children with ASD can complete set-shifting tasks, they require greater reliance on frontal regions, reflecting reduced efficiency in their cognitive control.

At the brain network level, the “Triple network model” proposed by Menon (13) provides a highly influential theoretical framework for understanding the pathophysiology of ASD and various other psychiatric disorders. This model posits that the dynamic imbalance among the salience network (SN), the central executive network (CEN), and the default mode network (DMN) is the core mechanism leading to cognitive and affective dysfunction (13). Research on ASD has widely corroborated this model. A study by Aggarwal and Gupta (14) showed that patients with ASD have abnormal functional connectivity in multiple functional brain networks, such as the cognitive control and default mode networks. Specifically, they exhibit under-connectivity within the cognitive control network, an abnormality that correlates with clinical symptoms. Similarly, Yoon et al. (12), using independent component analysis, also found under-connectivity in the cognitive control network of children with ASD, but hyper-connectivity between subcortical, visual, and somatomotor networks. These studies suggest that the atypical neurodevelopment in ASD manifests as varied patterns of connectivity abnormalities across different brain functional regions.

Despite the growing body of literature on cognitive control in ASD, there is currently a lack of comprehensive bibliometric analyses that map the knowledge structure, developmental trajectory, research hotspots, and future trends of this field. Bibliometric analysis, through the systematic statistical and visual processing of published literature, can intuitively reveal the academic landscape and evolutionary path of a research area. Therefore, this study utilizes bibliometric methods to conduct a comprehensive analysis of the neuroimaging literature on cognitive control in ASD, aiming to: 1) quantitatively describe the developmental history and overall trends of the field; 2) identify core research forces, including the most influential authors, institutions, and countries; 3) reveal research hotspots, the knowledge base, and thematic evolution through keyword and co-citation analysis; and 4) based on the analysis results, discuss the limitations of current research and prospect future directions, providing a reference for subsequent studies.

## 2 Materials and methods

### 2.1 Data source and search strategy

The data for this study were sourced from the Web of Science Core Collection and Scopus. These two databases were selected because they are recognized as the most authoritative multidisciplinary citation databases offering comprehensive journal coverage (15). Although PubMed/MEDLINE is highly relevant for medical research, it was not included as a primary data source because it functions primarily as a search engine for biomedical literature and does not provide the citation indexing (specifically, the “Cited References” metadata) required for the co-citation and bibliometric network analyses performed in this study (16). We acknowledge that some clinically oriented ASD papers may be indexed only in PubMed/MEDLINE and are therefore not captured in our dataset, which may introduce coverage bias.

The search period was set from January 1, 2000, to December 31, 2025. We selected 2000 as the start year to capture the modern, database-indexed era of ASD neuroimaging research and to align with the broader uptake of MRI-based neuroimaging methods in the early 2000s. The search strategy was constructed based on Medical Subject Headings (MeSH) and keywords identified from key prior studies. It revolves around three core concepts: ASD, cognitive control, and neuroimaging. Concept 1 (ASD): (“autism” OR “autism spectrum disorder” OR “autistic spectrum disorder” OR “ASD” OR “autistic disorder” OR “Asperger syndrome” OR “pervasive developmental disorder”); Concept 2 (Cognitive Control): (“cognitive control” OR “executive function” OR “executive functioning” OR “working memory” OR “inhibitory control” OR “inhibition” OR “cognitive flexibility” OR “set shifting” OR “cognitive shifting” OR “attention control”); Concept 3 (Neuroimaging): (“neuroimaging” OR “brain imaging” OR “functional magnetic resonance imaging” OR “fMRI” OR “structural MRI” OR “diffusion tensor imaging” OR “DTI” OR “positron emission tomography” OR “PET” OR “magnetoencephalography” OR “MEG” OR “electroencephalography” OR “EEG”). We note that some task-based fMRI studies relevant to cognitive control (e.g., Stop-Signal, Flanker, Stroop, n-back) may not explicitly label these constructs in titles/abstracts/keywords and could therefore be under-retrieved by a term-based Concept 2 query.

To validate the search strategy, we performed a pragmatic recall (sanity) check by verifying the retrieval of ten seminal articles known to be central to this field; all were successfully identified. We acknowledge that this is a limited benchmark relative to the full corpus and does not constitute a formal sensitivity analysis. The detailed search syntax for both databases is provided in **Supplementary Table 1**. The final search query combined all concepts: (Concept 1) AND (Concept 2) AND (Concept 3), and was limited to document types ‘article’ or ‘review article’ and language ‘English’.

### 2.2 Literature screening

**Figure 1:**
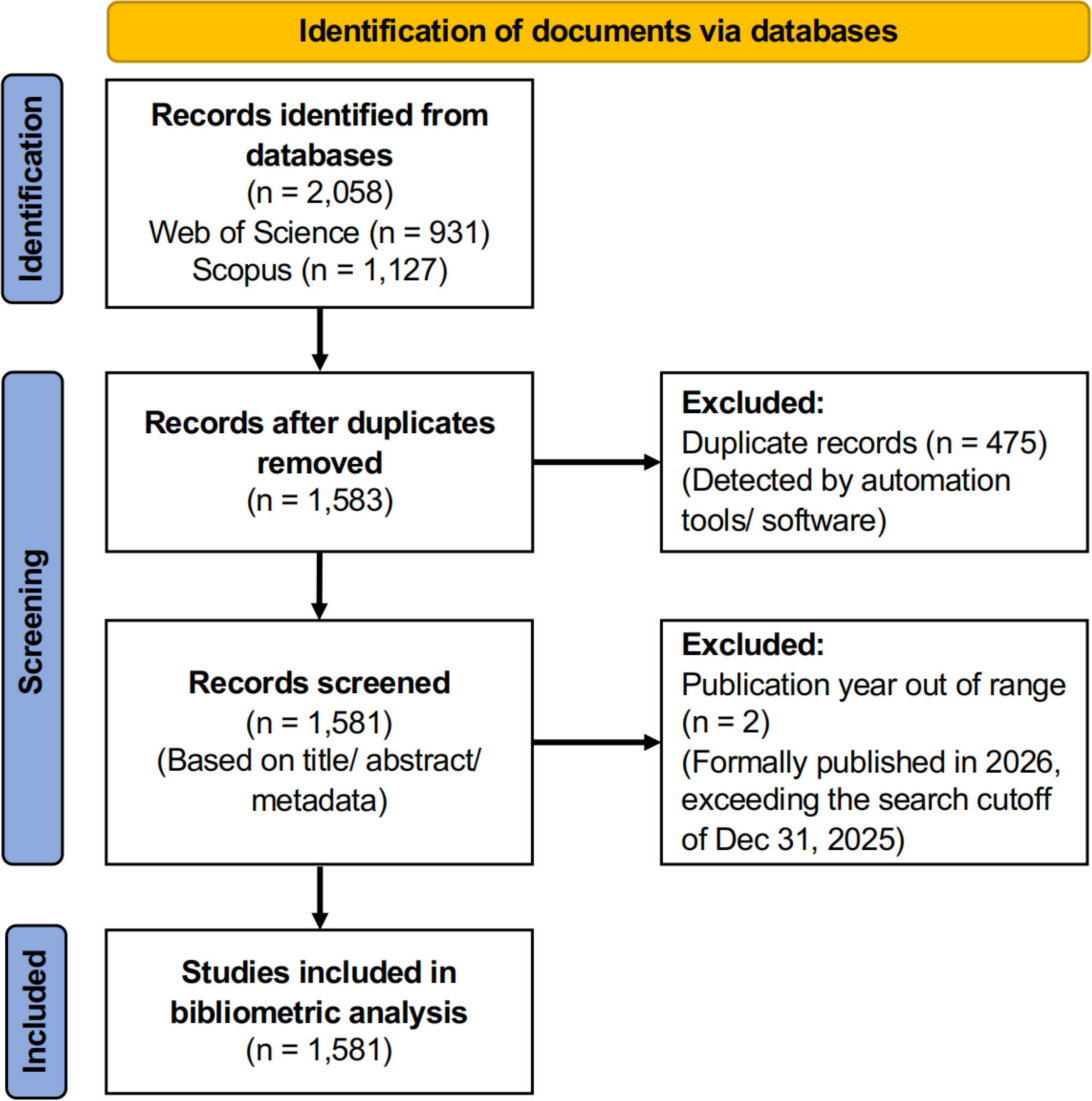
PRISMA 2020 flow diagram of the literature selection process. Screening was conducted at the metadata level (title/abstract/records) using database filters (timeframe, language, document type) and manual checks to exclude clearly irrelevant records and non-eligible document types. Full-text screening was not performed because this study is a bibliometric metadata analysis. Two records were excluded post-deduplication because their formal publication year was 2026, exceeding the cutoff date (December 31, 2025).

The literature retrieved from the two databases was imported into a reference management software for merging and removal of duplicates. Subsequently, titles and abstracts were manually screened to exclude conference abstracts, book reviews, and articles clearly irrelevant to the topic. To ensure the reliability and rigor of this manual selection process, a second researcher (CHJ) independently re-examined a random sample of 20% of the processed records (n = 316). The inter-rater agreement was calculated at 98.73%, confirming the consistency of the screening criteria. In addition, two records were excluded after deduplication because their formal publication year was 2026, which exceeded the predefined cutoff date (December 31, 2025). After this rigorous screening process, a final set of 1,581 articles was included for analysis (**Figure 1**). The reporting of this study follows the PRISMA 2020 guidelines where applicable to bibliometric analyses. Specific adaptations and exclusions of PRISMA items (e.g., omission of risk of bias assessment and meta-analytic synthesis) are detailed in the PRISMA-Bibliometric Compliance Table provided in **Supplementary Table 2**.

### 2.3 Risk of bias assessment and protocol

Given that this study is a bibliometric analysis (mapping review) focusing on the quantitative characteristics of metadata, distinct from a standard systematic review that synthesizes qualitative outcomes of clinical trials, a formal assessment of the risk of bias (e.g., using the Cochrane Risk of Bias Tool) was not applicable. The quality of the included data relies on the indexing standards of the Web of Science and Scopus databases. The methodological rigor of this bibliometric analysis is ensured through comprehensive database coverage, systematic search strategies, and transparent reporting of all analytical procedures.

### 2.4 Data analysis

This study employed VOSviewer (1.6.20) and the Bibliometrix package (4.3.0) in R as the primary analysis tools. VOSviewer was used to construct and visualize scientific knowledge maps, including keyword co-occurrence networks, author co-citation networks, and country collaboration networks, to reveal research hotspots and the thematic structure of the field (17). The Bibliometrix package was used for quantitative bibliometric analysis, such as publication trends, core author analysis, calculation of impact metrics like Lotka’s Law, and thematic evolution analysis (18).

## 3 Results

### 3.1 Overall trends in literature

A total of 1,581 documents published between 2000 and 2025 were included in this study. These articles were published in 458 journals by 7,479 authors. The average annual growth rate of publications was 21.61%, with an average document age of 7.86 years and an average of 57.70 citations per document. In terms of keywords, 8,469 keywords plus and 3,264 author’s keywords were identified. The field is highly collaborative, with an average of 6.81 co-authors per document and an international collaboration rate of 17.71%. Detailed information is provided in **Table 1**.

**Table 1:** Main information about the data (n = 1581).

Description	Results
Timespan	2000~2025
Journals	458
Documents	1581
Annual growth rate %	21.61
Document average age (years)	7.86
Average citations per document	57.70
Keywords plus	8469
Author’s keywords	3264
Authors	7479
Authors of single-authored docs	66
Single-authored documents	67
Co-authors per document	6.81
International co-authorships %	17.71

The volume of literature on neuroimaging of cognitive control in ASD shows a significant overall growth trend, which can be divided into three phases. Nascent phase (2000-2005): The average annual publication volume was less than 10, indicating the field was in its early exploratory stage. Stable growth phase (2006-2011): The average annual publication volume increased to approximately 30, with steadily growing academic interest. Rapid growth phase (2012-2025): The publication volume surged dramatically, reaching a peak of 140 articles in 2022 (see **Supplementary Figure 1**). This explosive growth is closely linked to the maturation and widespread adoption of neuroimaging technologies like fMRI, as well as the broad acceptance of brain network research within the academic community.

### 3.2 Main research forces

**Figure 2:**
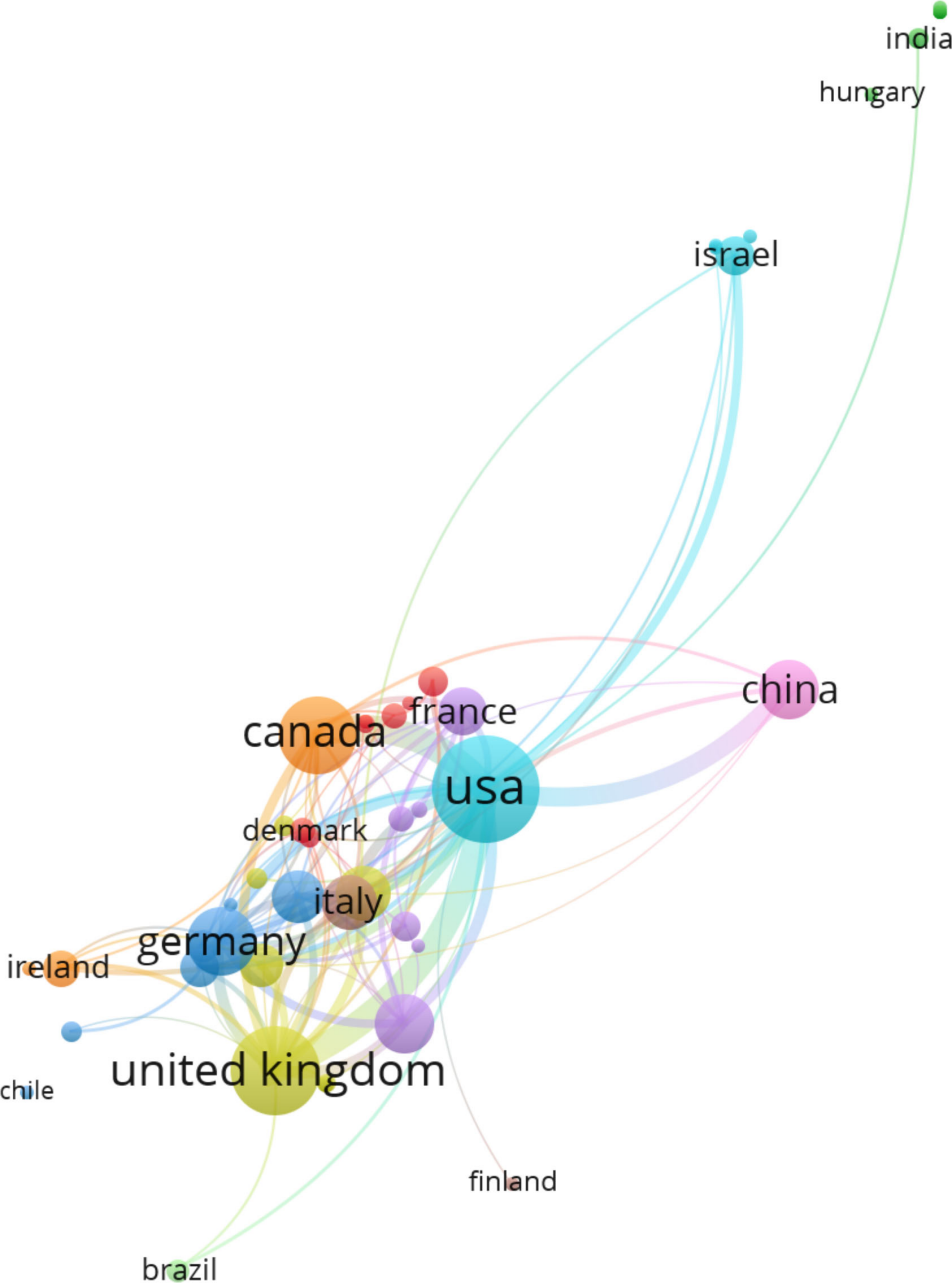
Country/region collaboration network map. The size of the nodes represents the number of publications. Nodes of the same color indicate closer collaboration clusters, and the lines represent cooperation between countries.

The analysis reveals a tight-knit international collaboration network, which is crucial for fostering the exchange of ideas, expertise, and resources (19). At the national level, the United States (553 articles) holds a dominant position in both academic output and network centrality. China (149 articles) and the United Kingdom (121 articles) form the second tier of research (see **Supplementary Figure 2**). The collaboration network map (**Figure 2**) further illustrates an international cooperative structure centered around the USA, connecting it with the UK, Germany, Canada, and China.

At the institutional level, the University of Toronto (206 articles), the University of California System (160 articles), and the University of London (155 articles) are the most productive research institutions globally. The distribution of journals follows Bradford’s Law, meaning a few core journals published the majority of relevant literature. The top 10 core journals published 377 articles, accounting for 23.85% of the total.

Regarding academic impact and status, Autism Research ranked first in publication volume (52 articles) and demonstrated the highest cumulative impact, as evidenced by its leading G-index (**Supplementary Figure 5**). This highlights its central role in disseminating high-quality, specialized research on ASD mechanisms. Notably, the core journal list reflects a dual-contribution pattern in the field. On one hand, domain-specific journals like Autism Research and the Journal of Autism and Developmental Disorders (42 articles) provide the clinical and behavioral context essential for understanding cognitive control deficits. On the other hand, high-impact neuroscience journals such as NeuroImage (44 articles), Cerebral Cortex (34 articles), and Neuroscience and Biobehavioral Reviews (26 articles) have made significant contributions by establishing rigorous neuroimaging methodologies (e.g., fMRI connectivity analysis). The prominence of these journals underscores that the advancement of this field relies heavily on the intersection of clinical psychiatry and advanced neuroimaging techniques. For detailed rankings of institutions and journals, see **Supplementary Table 3** and **Supplementary Figures 3–S5**.

### 3.3 Author and document impact analysis

**Figure 3:**
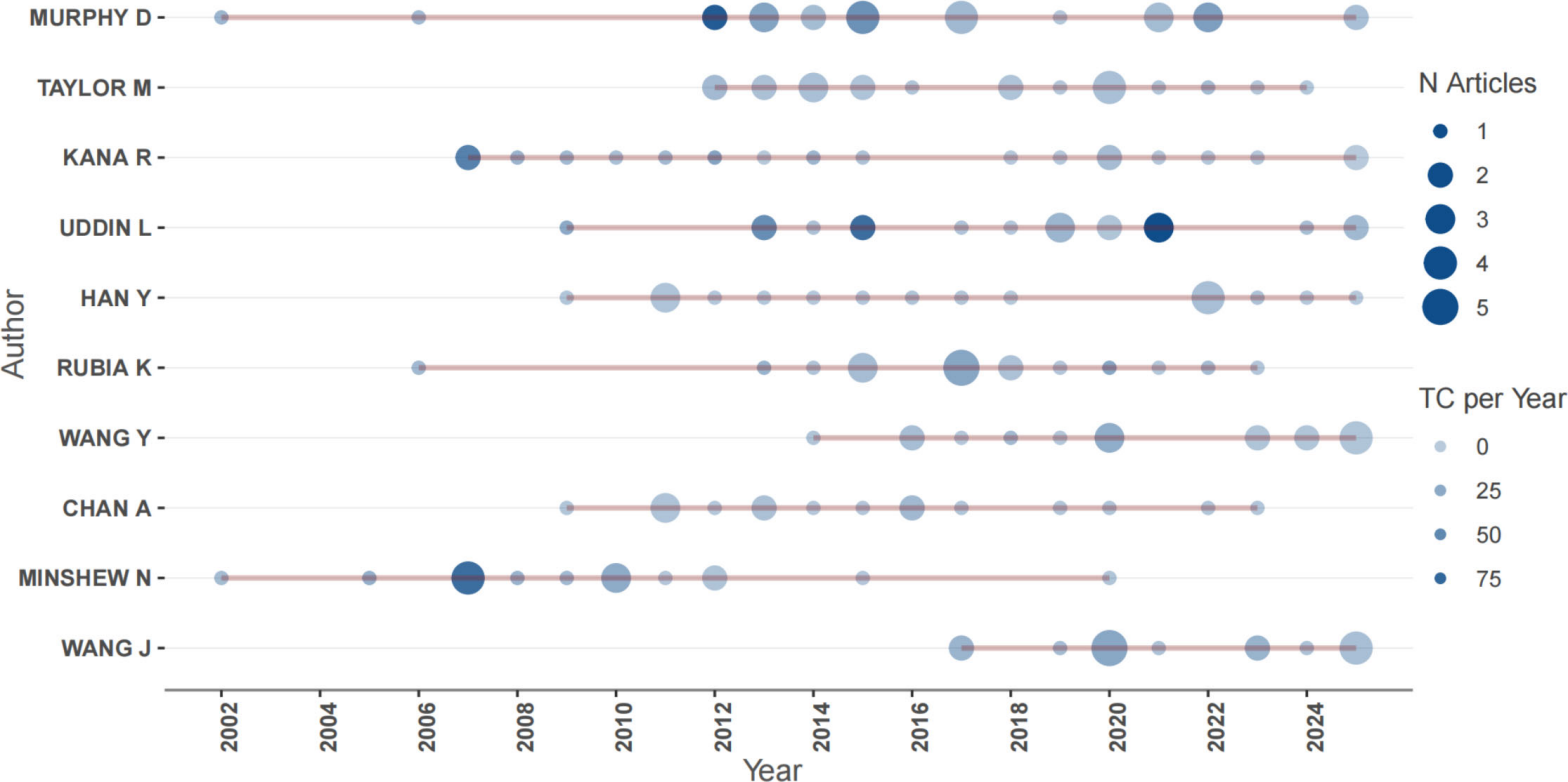
Dynamic publication volume and citations of the top 10 authors. The x-axis and y-axis represent the year and authors, respectively. The larger the node, the more articles published; the darker the node color, the higher the citation count.

Based on publication volume, the top four authors are Murphy, D.G.M. (26 articles), Taylor, M.J. (21), Kana, R. (19), and Uddin, L.Q. (19). Notably, Murphy, D.G.M. has been producing high-impact research since 2002; his 26 articles have garnered 3,313 citations, and his H-index of 20, demonstrating his long-term leadership in the field (**Figure 3**; **Supplementary Table 4**). However, Lotka’s Law analysis indicated that 79.54% of authors contributed only one paper, revealing a typical research collaboration pattern dominated by a few core authors (see **Supplementary Figure 6**). The author co-citation network is also highly structured, indicating a concentrated intellectual base and several tightly connected knowledge communities (**Supplementary Figure 7**). In this map, a small number of highly influential authors form the core of the network and bridge multiple clusters, reflecting the multidisciplinary integration essential for advancing the field.

Analysis of highly cited documents reveals the pivotal theoretical and mechanistic shifts in this domain. The most cited paper, Menon (13) (3,362 citations), established a foundational theoretical framework by proposing the “Triple Network Model.” This work posited that psychopathology, including autism, stems from deficits in the Salience Network’s ability to dynamically switch between the DMN and the CEN, thereby defining the macro-scale network perspective. Complementing this, Uhlhaas and Singer (20) and Wang (21) shifted the focus toward physiological mechanisms. Their works highlighted how abnormal neural synchrony and excitation-inhibition (E-I) imbalances disrupt long-range connectivity, providing a mechanistic explanation for the “disconnectivity” observed in ASD. Furthermore, Fair et al. (22) made a transformative contribution by characterizing the developmental trajectory of the DMN, demonstrating its transition from a sparsely connected state in childhood to a cohesive network in adulthood. This finding provided a critical baseline for identifying developmental deviations in children with ASD (see **Supplementary Figure 8**).

### 3.4 Thematic evolution and research hotspots

**Figure 4:**
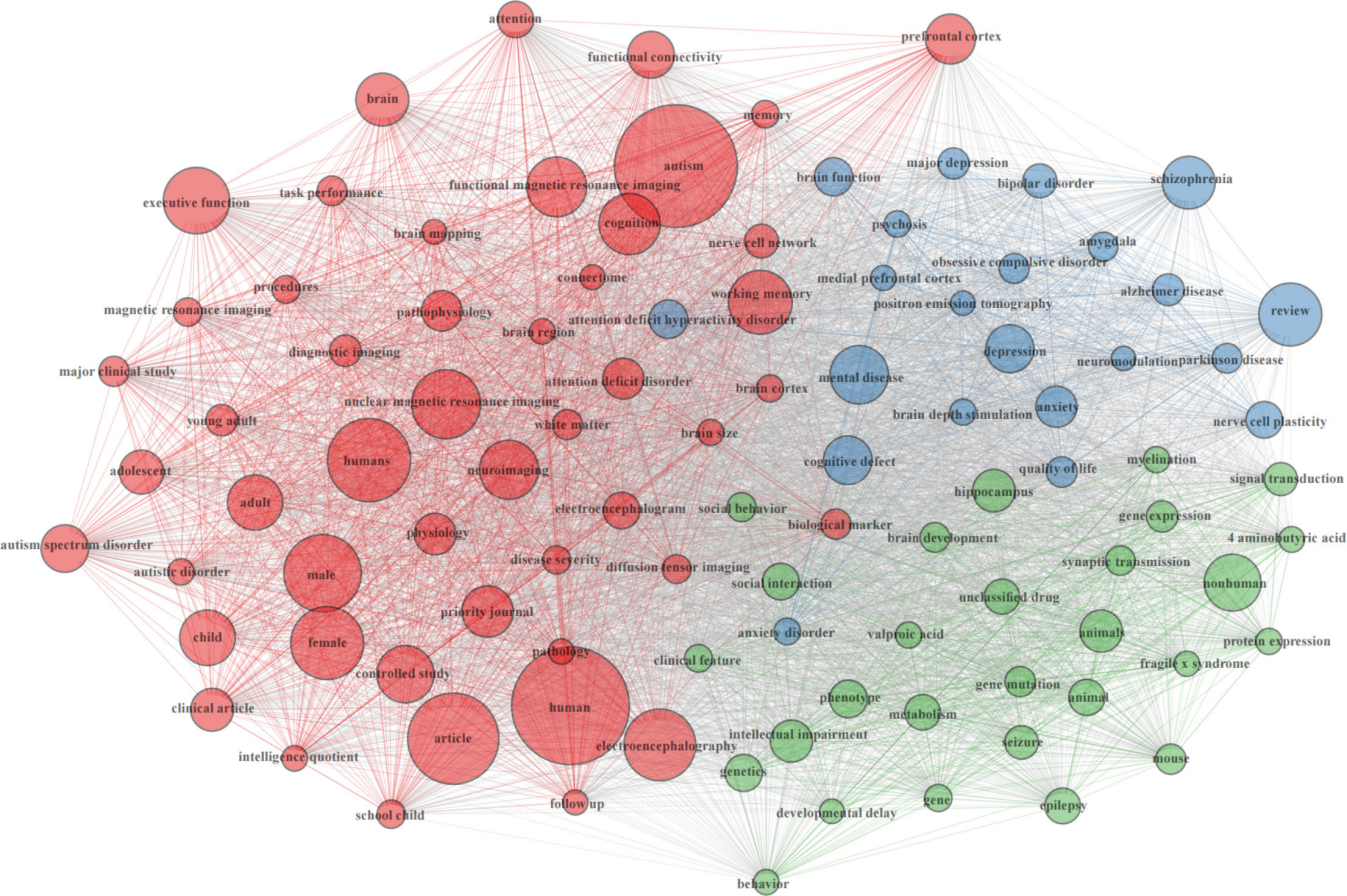
Keyword co-occurrence network map.

Keyword co-occurrence analysis revealed the main hotspots and structure of the research. Among the most prominent and frequently recurring keywords, “autism,” “executive function,” “functional connectivity,” and “fMRI” show consistently high usage and clear temporal growth patterns (see **Supplementary Figure 9**). Notably, the timing of thematic shifts (e.g., keyword centrality) may precede the rapid increase in publication volume; therefore, the “since 2010” pattern below refers to network centrality rather than annual output growth. Notably, the centrality of “functional connectivity” has risen rapidly since 2010, becoming the most critical node connecting different research themes (e.g., “attention,” “default mode network”). This clearly indicates a paradigm shift from a “functional localization” mindset, focused on isolated brain regions, to a “network connectivity” mindset that explores the synergistic work between brain regions (**Figure 4**).

**Figure 5:**
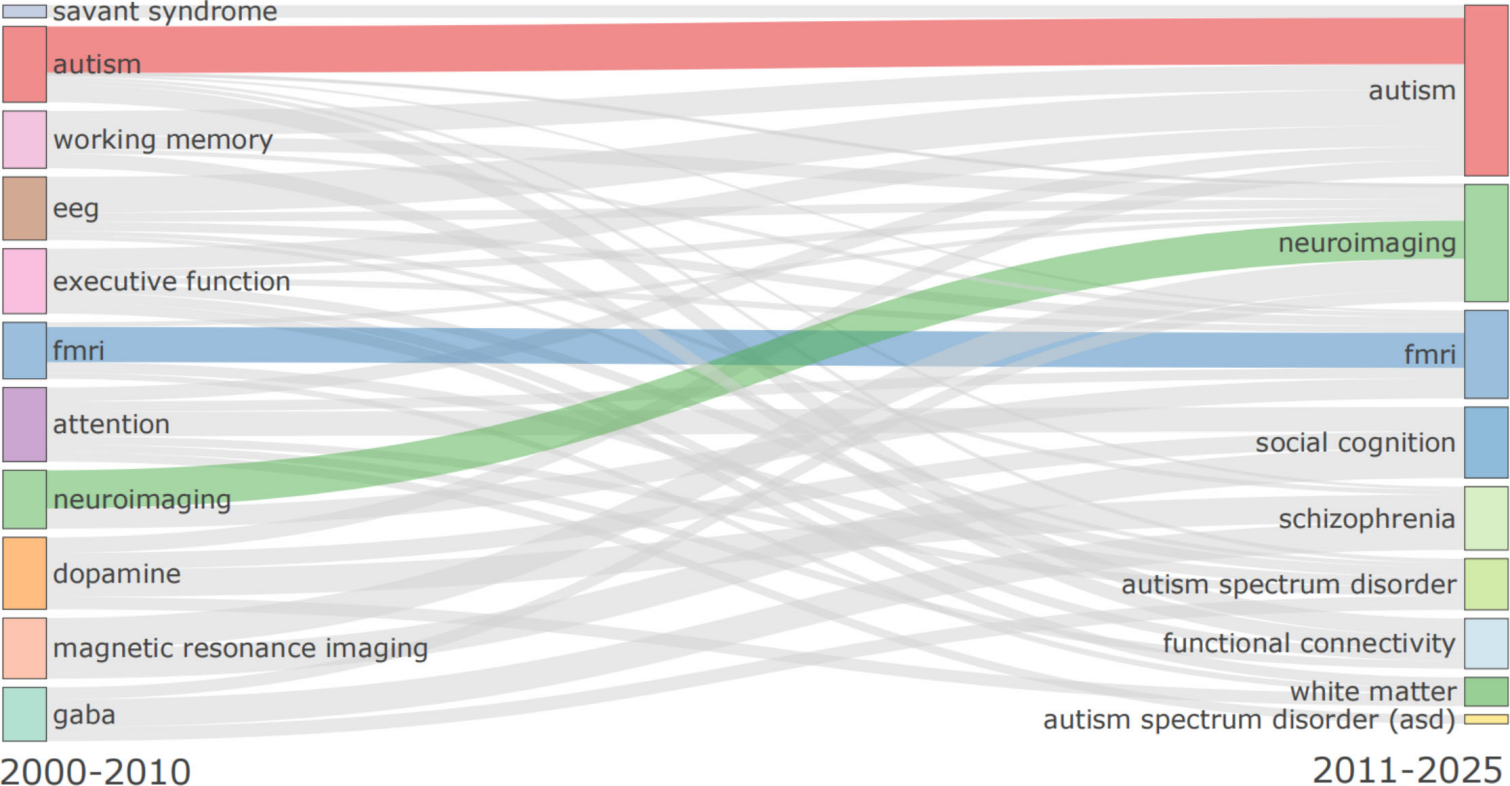
Thematic evolution map.

The thematic evolution analysis (**Figure 5**) reveals a clear shift in research emphasis from core cognitive constructs toward network- and method-oriented topics. During 2000–2010, themes largely centered on higher-order cognition (e.g., “working memory,” “executive function”) and modality-specific terms (e.g., “EEG”). In the 2011–2025 period, a network-science paradigm became increasingly prominent: “functional connectivity” emerged as a major integrative theme linking earlier cognitive topics with neuroimaging-driven investigations. Notably, several terms remain concurrently used rather than fully consolidated (e.g., “autism” and “autism spectrum disorder,” as well as variant labels such as “ASD”), reflecting ongoing terminological heterogeneity in the literature. Likewise, topics such as “white matter” continue to appear as distinct themes alongside broader labels (e.g., “neuroimaging”), suggesting parallel rather than fully merged thematic trajectories.

**Figure 6:**
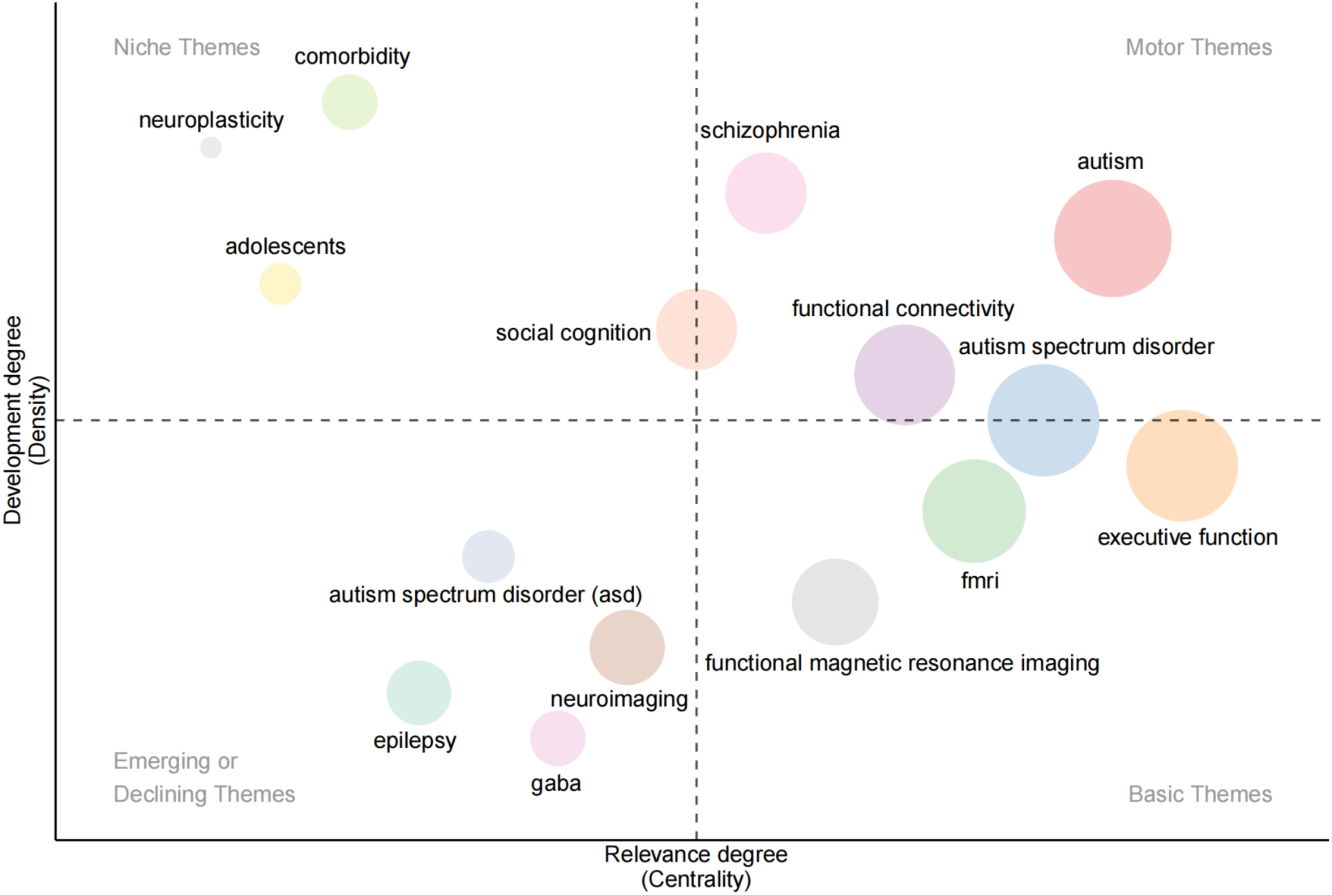
Thematic map. The thematic map illustrates the thematic structure of neuroimaging research on cognitive control in ASD. The size of the bubbles is proportional to the keyword frequency. The x-axis (centrality) measures the correlation between themes, while the y-axis (density) measures the maturity of a theme.

Through the thematic map (**Figure 6**), we further analyzed the centrality (the importance and relevance of a theme within the field) and density (its internal developmental maturity), thereby revealing the field’s knowledge structure (see **Supplementary Table 5** for details). The basic themes (high centrality, low density) include foundational cognitive and methodological topics such as “executive function” and imaging-related clusters (e.g., “fMRI” and “functional magnetic resonance imaging”), which serve as cornerstones connecting multiple research branches. In contrast, “autism,” “functional connectivity,” and “schizophrenia” constitute motor/driving themes (high centrality, high density), representing mature and influential research directions, consistent with the recent emphasis on network neuroscience and transdiagnostic comparisons. Several specialized themes appear as niche themes (low centrality, high density), such as “comorbidity,” “adolescents,” and “neuroplasticity,” indicating well-developed but relatively less connected subfields. Notably, “social cognition” occupies an intermediate position with both niche-like maturity and meaningful links to the broader network of topics (**Supplementary Table 5**), reflecting its role as both a specialized and cross-cutting theme in ASD-related neuroimaging research. Finally, themes in the emerging or declining quadrant (low centrality, low density) include “GABA” and “epilepsy,” as well as more general labels such as “neuroimaging” and certain ASD variant terms (e.g., “autism spectrum disorder (ASD)”), which may partly reflect terminological fragmentation and the fact that broad umbrella terms can form relatively diffuse clusters compared with more specific, method- or mechanism-focused topics.

## 4 Discussion

Through bibliometric methods, this study has systematically mapped the knowledge landscape of neuroimaging research on cognitive control in ASD over the past two decades. The results not only quantify the exponential growth of the field but also profoundly reveal the evolution of its research paradigms, core theories, and future trends.

### 4.1 Evolution and growth drivers of the field

This study first reveals the macro-level trend of exponential growth in the field over the past two decades, with its developmental course clearly divisible into three stages: the nascent exploratory phase (2000-2005), the stable growth phase (2006-2011), and the rapid growth phase (2012-2025). In the nascent phase, research primarily focused on the behavioral manifestations and clinical features of ASD (23). For example, simple cognitive tasks were used to identify difficulties in areas like inhibition and cognitive flexibility in individuals with ASD. However, limited by the technologies and methods of the time, these early studies had a limited understanding of the neural mechanisms underlying these cognitive control deficits.

The sharp increase in annual publications after 2012 marks the field’s entry into an “explosive” growth phase. This growth curve is highly correlated with the innovation of neuroimaging technologies, particularly the widespread application of functional magnetic resonance imaging (fMRI). As observed by Hernandez et al. (24) with a similar trend in the broader neuroimaging field, the maturation and popularization of technology serve as a key catalyst for exponential research growth. Technologies like fMRI enable researchers to non-invasively observe real-time brain activity in individuals with ASD while they perform cognitive control tasks, providing unprecedented tools to explore their neural mechanisms and thereby shifting the research from phenomenological description to the deeper exploration of underlying mechanisms.

### 4.2 Paradigm shift: from functional localization to network dysregulation

One of the most central findings of this study is the profound paradigm shift within the field. As our keyword analysis shows, the centrality of “functional connectivity” has risen sharply since 2010, becoming the hub connecting all key themes. This shift represents a transition from early attempts to localize functional abnormalities in specific brain regions to exploring the coordination and dysregulation among large-scale brain networks. Our co-citation analysis identifies the “Triple network model” proposed by Menon (13) as the foundational theoretical pillar. This framework posits that the SN—anchored in the anterior insula and anterior cingulate cortex—acts as a dynamic switch, regulating the transition between the internally oriented DMN and the externally oriented CEN. Menon hypothesized that dysfunction in this switching mechanism leads to the core cognitive and affective deficits observed in psychopathology.

Crucially, this network perspective has moved beyond theoretical abstraction to drive empirical discoveries regarding specific ASD phenotypes. Grounded in Menon’s framework, Uddin et al. (25) challenged the prevailing “underconnectivity” theory by demonstrating that children with ASD actually exhibit intrinsic hyperconnectivity within the Salience Network. They found that this excessive connectivity predicted the severity of restricted and repetitive behaviors, suggesting that an “over-attribution of salience” to irrelevant stimuli may underlie the sensory and behavioral rigidity in childhood autism. Extending this investigation to network interactions, Abbott et al. (26) revealed that network dysfunction in ASD is characterized by impaired network segregation. Specifically, they observed atypical overconnectivity between the DMN and CEN (implying a failure to decouple these competing networks), alongside underconnectivity within the SN. Importantly, Abbott et al. linked these specific connectivity profiles to distinct behavioral outcomes, associating reduced SN integrity with sensory and socio-communicative deficits, while poor DMN-CEN segregation correlated with executive dysfunction.

However, the juxtaposition of “hyperconnectivity” in children (25) and “underconnectivity” in older cohorts (26) highlights the necessity of a developmental perspective. Nomi and Uddin (27) explicitly addressed this by identifying a “developmental flip” in ASD neural circuitry. Their large-scale analysis demonstrated that the autistic brain trajectory is not static; rather, it transitions from widespread hyperconnectivity in childhood to hypoconnectivity in adolescence and adulthood. This synthesis suggests that while Menon’s Triple Network Model correctly identifies the locus of dysfunction (the SN-DMN-CEN interaction), the nature of the dysregulation is dynamic. Thus, the field is now evolving from simply identifying static network deficits to mapping their complex, non-linear developmental trajectories across the lifespan.

### 4.3 Broadening perspectives: from a single disorder to transdiagnostic research

The thematic evolution analysis reveals the recent emergence of “transdiagnostic” research, another significant milestone in the field’s development. ASD shares a high degree of overlap with other neurodevelopmental disorders like ADHD in clinical symptoms, genetic factors, and cognitive deficits (28). The traditional research paradigm based on diagnostic categories struggles to explain this commonality. Transdiagnostic research, in contrast, seeks to move beyond phenomenological descriptions by using data-driven methods like machine learning to identify biologically meaningful subtypes that cut across traditional diagnostic boundaries from large-scale neuroimaging data (29–31). As our thematic map shows, the emergence of “schizophrenia” as a motor/driving theme also reflects researchers’ efforts to deepen their understanding of ASD through comparisons with other psychiatric disorders. This trend not only helps to uncover shared pathophysiological mechanisms across different diseases but also opens up possibilities for achieving “precision medicine” based on individual biological characteristics. Specifically, the prominent positioning of ‘schizophrenia’ as a motor theme (high centrality and density) in our thematic map (**Figure 6**) is not merely a bibliometric artefact, but reflects a deep mechanistic pivot in the field. Researchers are increasingly leveraging the well-documented network dysfunctions in schizophrenia—such as excitation-inhibition imbalances—as a mature theoretical template to decode similar socio-cognitive deficits in ASD. This cross-pollination suggests that the field is moving away from isolating ASD-specific biomarkers toward identifying shared neural dimensions of cognitive rigidity across the broader psychosis-autism spectrum.

### 4.4 Future outlook: integration, dynamics, and clinical translation

Based on the bibliometric trends and emerging hotspots identified in this study, future research should move beyond descriptive mapping to address mechanistic and translational questions. Importantly, our thematic map suggests that some directions are already well-established and field-driving (e.g., “functional connectivity” as a motor theme), whereas others remain comparatively peripheral or less consolidated (e.g., neurochemical terms such as “GABA/glutamate” in the emerging/declining quadrant; **Supplementary Table 5**). We therefore outline three directions while distinguishing between mature versus still-developing trajectories, and we contextualize them with recent related reviews and meta-analyses (32–35).

First, the emergence of neurochemical themes, such as GABAergic and glutamatergic signaling, highlights a critical shift toward understanding the molecular underpinnings of ASD. However, current neuroimaging literature is frequently plagued by issues of low reproducibility and site-specific biases. Relying on single-modality imaging has yielded inconsistent findings due to small sample sizes, substantial phenotypic heterogeneity, and methodological variability (36). To resolve this, future research must prioritize the deep integration of multimodal data, fusing functional and structural metrics with neurochemical profiles obtained via 1H-MRS or PET (37). Furthermore, novel techniques like DTI-ALPS offer promising avenues to investigate the glymphatic system, potentially linking neuroinflammation and metabolic waste clearance to structural anomalies (38). A significant challenge ahead lies in developing robust computational frameworks capable of aligning data with vastly different temporal and spatial resolutions to construct a holistic neuropathological model.

Second, the field must move beyond static functional connectivity to capture the brain’s rapid reconfiguration. Traditional static analyses largely overlook the temporal instability of neural networks and are susceptible to motion artifacts, which are particularly problematic in pediatric ASD populations. Investigating dynamic functional connectivity metrics, such as state transitions and dwell times, offers a more nuanced understanding of the cognitive rigidity characteristic of ASD (4). This dynamic perspective is particularly vital for characterizing the “developmental flip” in connectivity patterns—from childhood hyper-connectivity to adult hypo-connectivity—and understanding how pubertal hormones influence network reorganization during the critical period of adolescence. Nevertheless, distinguishing true neural flexibility from physiological noise remains a methodological bottleneck that requires more rigorous acquisition protocols and multi-site harmonization strategies to mitigate scanner variability.

Finally, a critical imperative is bridging the gap between group-level biomarkers and individual clinical applications. The heterogeneity of ASD necessitates a shift toward mechanism-based stratification rather than “one-size-fits-all” treatments. For instance, neuroimaging evidence of glutamatergic dysregulation has directly informed the design of randomized controlled trials for agents like memantine, where imaging serves not only as a diagnostic tool but as a secondary endpoint to validate target engagement in fronto-striatal circuits (39). Future efforts should focus on using these multimodal and dynamic biomarkers to stratify patients into biological subgroups, thereby predicting individual responses to targeted pharmacological or behavioral interventions.

### 4.5 Limitations

Although this study employed various bibliometric analysis methods to reveal the knowledge structure and evolutionary trajectory of the field, several limitations should be acknowledged to provide a balanced interpretation of the results.

First, regarding data sources and indexing bias. The literature search was confined to the Web of Science and Scopus databases. While these platforms are considered the gold standards for bibliometric analysis due to their high-quality metadata and rigorous citation linkages, they are not free from systematic biases. As noted by Mongeon and Paul-Hus (15), both databases exhibit a distinct “Anglophone bias” and tend to under-represent journals from non-Western regions and in non-English languages. Consequently, our analysis may underestimate the contributions of institutions and authors from emerging regions who publish primarily in local or regional vernacular journals not indexed in these major platforms.

Second, regarding the sensitivity of network topology. The database selection directly influences the construction of collaboration and citation networks. The networks presented in this study primarily reflect the “core” international academic community. However, peripheral networks formed within specific linguistic or regional clusters might be omitted due to the aforementioned indexing coverage limitations. Therefore, the centrality measures of certain non-Western institutions or authors might be lower in our results than their actual influence within their local academic ecosystems. While combining WoS and Scopus improves coverage compared to using a single database, future studies could employ regional databases or altmetrics to validate these findings and capture a more comprehensive global picture. In addition, author-level collaboration metrics may be inflated by large consortium publications, and high collaborator counts should not be over-interpreted as a field-specific property without considering authorship practices.

Third, regarding the depth of analysis. Bibliometric analysis focuses on the external characteristics of literature (such as citations and keywords) and cannot provide an in-depth qualitative assessment of the research content quality, the rigor of experimental designs, or the reliability of conclusions. High citation counts do not always equate to high scientific validity.

Finally, regarding the time lag. As a rapidly developing field, new breakthrough research may have emerged after the data collection cutoff date for this study. The findings presented here represent a snapshot of the field’s development up to that point.

## 5 Conclusion

Through a bibliometric analysis of 1,581 articles from 2000 to 2025, this study systematically mapped the neuroimaging research landscape on cognitive control in ASD. The results demonstrate an exponential growth of the field over the past two decades and a profound paradigm shift from focusing on functional abnormalities in isolated brain regions to investigating the dysregulation of large-scale brain network connectivity. “Functional connectivity” and the “Triple Network Model” have emerged as central organizing frameworks of the knowledge base. Concurrently, the rise of transdiagnostic research indicates a movement toward integrative approaches that compare ASD with other neurodevelopmental and psychiatric conditions. Future work is expected to further advance via multimodal neuroimaging, machine learning, and interdisciplinary collaboration, thereby supporting earlier screening and more personalized interventions.

## Data Availability

The original contributions presented in the study are included in the article/**Supplementary Material**. Further inquiries can be directed to the corresponding author.

## References

[1] American Psychiatric Association . Diagnostic and statistical manual of mental disorders: DSM-5™. 5th ed. Arlington, VA: American Psychiatric Publishing, Inc (2013). doi: 10.1176/appi.books.9780890425596

[2] Maenner MJ Warren Z Williams AR Amoakohene E Bakian AV Bilder DA . Prevalence and characteristics of autism spectrum disorder among children aged 8 years — autism and developmental disabilities monitoring network, 11 sites, United State. MMWR Surveillance Summaries. (2023) 72:1–14. doi: 10.15585/mmwr.ss7202a1. PMID: 36952288 PMC10042614

[3] Maenner MJ Rice CE Arneson CL Cunniff C Schieve LA Carpenter LA . Potential impact of DSM-5 criteria on autism spectrum disorder prevalence estimates. JAMA Psychiatry. (2014) 71:292–300. doi: 10.1001/jamapsychiatry.2013.3893. PMID: 24452504 PMC4041577

[4] Uddin LQ . Brain mechanisms supporting flexible cognition and behavior in adolescents with autism spectrum disorder. Biol Psychiatry. (2021) 89:172–83. doi: 10.1016/j.biopsych.2020.05.010. PMID: 32709415 PMC7677208

[5] Van Eylen L Boets B Steyaert J Wagemans J Noens I . Executive functioning in autism spectrum disorders: influence of task and sample characteristics and relation to symptom severity. Eur Child Adolesc Psychiatry. (2015) 24:1399–417. doi: 10.1007/s00787-015-0689-1. PMID: 25697266

[6] Conson M Mazzarella E Esposito D Grossi D Marino N Massagli A . Put myself into your place: embodied simulation and perspective taking in autism spectrum disorders. Autism Res. (2015) 8:454–66. doi: 10.1002/aur.1460. PMID: 25663550

[7] Pelphrey KA Shultz S Hudac CM Vander Wyk BC . Research review: constraining heterogeneity: the social brain and its development in autism spectrum disorder. J Child Psychol Psychiatry. (2011) 52:631–44. doi: 10.1111/j.1469-7610.2010.02349.x. PMID: 21244421 PMC3096715

[8] Yerys BE Antezana L Weinblatt R Jankowski KF Strang J Vaidya CJ . Neural correlates of set-shifting in children with autism. Autism Res. (2015) 8:386–97. doi: 10.1002/aur.1454. PMID: 25599972 PMC4508240

[9] Yerys BE Herrington JD . Multimodal imaging in autism: an early review of comprehensive neural circuit characterization. Curr Psychiatry Rep. (2014) 16:496. doi: 10.1007/s11920-014-0496-2. PMID: 25260934

[10] Ecker C Bookheimer SY Murphy DG . Neuroimaging in autism spectrum disorder: brain structure and function across the lifespan. Lancet Neurol. (2015) 14:1121–34. doi: 10.1016/s1474-4422(15)00050-2. PMID: 25891007

[11] Rane P Cochran D Hodge SM Haselgrove C Kennedy DN Frazier JA . Connectivity in autism: a review of MRI connectivity studies. Harv Rev Psychiatry. (2015) 23:223–44. doi: 10.1097/hrp.0000000000000072. PMID: 26146755 PMC5083037

[12] Yoon N Kim S Oh MR Kim M Lee JM Kim BN . Intrinsic network abnormalities in children with autism spectrum disorder: an independent component analysis. Brain Imaging Behav. (2024) 18:430–43. doi: 10.1007/s11682-024-00858-x. PMID: 38324235

[13] Menon V . Large-scale brain networks and psychopathology: a unifying triple network model. Trends Cognit Sci. (2011) 15:483–506. doi: 10.1016/j.tics.2011.08.003. PMID: 21908230

[14] Aggarwal P Gupta A . Multivariate graph learning for detecting aberrant connectivity of dynamic brain networks in autism. Med Image Anal. (2019) 56:11–25. doi: 10.1016/j.media.2019.05.007. PMID: 31150935

[15] Mongeon P Paul-Hus A . The journal coverage of Web of Science and Scopus: a comparative analysis. Scientometrics. (2016) 106:213–28. doi: 10.1007/s11192-015-1765-5. PMID: 30311153

[16] Falagas ME Pitsouni EI Malietzis GA Pappas G . Comparison of PubMed, Scopus, Web of Science, and Google Scholar: strengths and weaknesses. FASEB Journal: Off Publ Fed Am Societies For Exp Biol. (2008) 22:338–42. doi: 10.1096/fj.07-9492LSF. PMID: 17884971

[17] van Eck NJ Waltman L . Software survey: VOSviewer, a computer program for bibliometric mapping. Scientometrics. (2010) 84:523–38. doi: 10.1007/s11192-009-0146-3. PMID: 20585380 PMC2883932

[18] Aria M Cuccurullo C . Bibliometrix: an R-tool for comprehensive science mapping analysis. J Informetrics. (2017) 11:959–75. doi: 10.1016/j.joi.2017.08.007. PMID: 38826717

[19] Chen K Zhang Y Fu X . International research collaboration: an emerging domain of innovation studies? Res Policy. (2019) 48:149–68. doi: 10.1016/j.respol.2018.08.005. PMID: 38826717

[20] Uhlhaas PJ Singer W . Neural synchrony in brain disorders: relevance for cognitive dysfunctions and pathophysiology. Neuron. (2006) 52:155–68. doi: 10.1016/j.neuron.2006.09.020. PMID: 17015233

[21] Wang XJ . Neurophysiological and computational principles of cortical rhythms in cognition. Physiol Rev. (2010) 90:1195–268. doi: 10.1152/physrev.00035.2008. PMID: 20664082 PMC2923921

[22] Fair DA Cohen AL Dosenbach NU Church JA Miezin FM Barch DM . The maturing architecture of the brain's default network. Proc Natl Acad Sci USA. (2008) 105:4028–32. doi: 10.1073/pnas.0800376105. PMID: 18322013 PMC2268790

[23] Minshew NJ Goldstein G Siegel DJ . Neuropsychologic functioning in autism: profile of a complex information processing disorder. J Int Neuropsychol Soc. (1997) 3:303–16. doi: 10.1017/s1355617797003032. PMID: 9260440

[24] Hernandez LM Rudie JD Green SA Bookheimer S Dapretto M . Neural signatures of autism spectrum disorders: insights into brain network dynamics. Neuropsychopharmacology. (2015) 40:171–89. doi: 10.1038/npp.2014.172. PMID: 25011468 PMC4262896

[25] Uddin LQ Supekar K Lynch CJ Khouzam A Phillips J Feinstein C . Salience network-based classification and prediction of symptom severity in children with autism. JAMA Psychiatry. (2013) 70:869–79. doi: 10.1001/jamapsychiatry.2013.104. PMID: 23803651 PMC3951904

[26] Abbott AE Nair A Keown CL Datko M Jahedi A Fishman I . Patterns of atypical functional connectivity and behavioral links in autism differ between default, salience, and executive networks. Cereb Cortex. (2016) 26:4034–45. doi: 10.1093/cercor/bhv191. PMID: 26351318 PMC5027998

[27] Nomi JS Uddin LQ . Developmental changes in large-scale network connectivity in autism. NeuroImage Clin. (2015) 7:732–41. doi: 10.1016/j.nicl.2015.02.024. PMID: 25844325 PMC4375789

[28] Liu A Lu Y Gong C Sun J Wang B Jiang Z . Bibliometric analysis of research themes and trends of the co-occurrence of autism and ADHD. Neuropsychiatr Dis Treat. (2023) 19:985–1002. doi: 10.2147/ndt.S404801. PMID: 37138730 PMC10149780

[29] Cordova M Shada K Demeter DV Doyle O Miranda-Dominguez O Perrone A . Heterogeneity of executive function revealed by a functional random forest approach across ADHD and ASD. NeuroImage Clin. (2020) 26:102245. doi: 10.1016/j.nicl.2020.102245. PMID: 32217469 PMC7109457

[30] Wang X Zhao K Yao L Fonzo GA Satterthwaite TD Rekik I . Delineating transdiagnostic subtypes in neurodevelopmental disorders via contrastive graph machine learning of brain connectivity patterns. In: bioRxiv (Cold Spring Harbor, NY: Cold Spring Harbor Laboratory) (2024). doi: 10.1101/2024.02.29.582790

[31] Zhang M Huang Y Jiao J Yuan D Hu X Yang P . Transdiagnostic symptom subtypes across autism spectrum disorders and attention deficit hyperactivity disorder: validated by measures of neurocognition and structural connectivity. BMC Psychiatry. (2022) 22:102. doi: 10.1186/s12888-022-03734-4. PMID: 35139813 PMC8827180

[32] Gkintoni E Panagioti M Vassilopoulos SP Nikolaou G Boutsinas B Vantarakis A . Leveraging AI-driven neuroimaging biomarkers for early detection and social function prediction in autism spectrum disorders: a systematic review. Healthcare (Basel Switzerland). (2025) 13:1776. doi: 10.3390/healthcare13151776. PMID: 40805809 PMC12346713

[33] Liu J Chen H Wang H Wang Z . Neural correlates of facial recognition deficits in autism spectrum disorder: a comprehensive review. Front Psychiatry. (2025) 15:1464142. doi: 10.3389/fpsyt.2024.1464142. PMID: 39834575 PMC11743606

[34] Nedungadi P Shah SM Stokes MA Kumar Nair V Moorkoth A Raman R . Mapping autism's research landscape: trends in autism screening and its alignment with sustainable development goals. Front Psychiatry. (2024) 14:1294254. doi: 10.3389/fpsyt.2023.1294254. PMID: 38361829 PMC10868528

[35] Wei L Zhou M Hu P Jia S Zhong S . Abnormal brain activation in autism spectrum disorder during negative emotion processing: a meta-analysis of functional neuroimaging studies. J Psychiatr Res. (2025) 185:1–10. doi: 10.1016/j.jpsychires.2025.03.032. PMID: 40138749

[36] Halliday AR Vucic SN Georges B LaRoche M Mendoza Pardo MA Swiggard LO . Heterogeneity and convergence across seven neuroimaging modalities: a review of the autism spectrum disorder literature. Front Psychiatry. (2024) 15:1474003. doi: 10.3389/fpsyt.2024.1474003. PMID: 39479591 PMC11521827

[37] Zürcher NR Bhanot A McDougle CJ Hooker JM . A systematic review of molecular imaging (PET and SPECT) in autism spectrum disorder: current state and future research opportunities. Neurosci Biobehav Rev. (2015) 52:56–73. doi: 10.1016/j.neubiorev.2015.02.002. PMID: 25684726

[38] Wang M Xu D Zhang L Jiang H . Application of multimodal MRI in the early diagnosis of autism spectrum disorders: a review. Diagnostics (Basel). (2023) 13:3027. doi: 10.3390/diagnostics13193027. PMID: 37835770 PMC10571992

[39] Häge A Banaschewski T Buitelaar JK Dijkhuizen RM Franke B Lythgoe DJ . Glutamatergic medication in the treatment of obsessive compulsive disorder (OCD) and autism spectrum disorder (ASD) - study protocol for a randomised controlled trial. Trials. (2016) 17:141. doi: 10.1186/s13063-016-1266-8. PMID: 26983548 PMC4794817

