# Supplementary material for: A bibliometric analysis of neuroimaging studies on cognitive control in autism spectrum disorder (2000–2025)
Source: Front Psychiatry. 2026 May 26;17:1765161. doi: 10.3389/fpsyt.2026.1765161 (PMC13246715; doi:10.3389/fpsyt.2026.1765161)
Supplement: Supplementary file 1 [file SupplementaryFile1.docx]

**Supplementary Materials**

**Figure S1.** Annual Publication Volume 2

**Figure S2.** Bar Chart of Collaboration by Major Countries/Regions 3

**Figure S3.** Dynamic Publication Volume of the Top 10 Journals 4

**Figure S4.** The Relationship Between Publishing Sources and the Number of Published Articles 5

**Figure S5.** H-Index and Its Derived Indices (G-Index, M-Index) of Publishing Sources 6

**Figure S6.** Lotka's Law Analysis of Publication Distribution 7

**Figure S7.** Author Co-citation Network 8

**Figure S8.** Highly Cited Documents 9

**Figure S9.** Keyword Frequency Over Time 10

**Table S1.** Detailed Search Queries for Web of Science and Scopus 11

**Table S2.** PRISMA 2020 Adaptation Checklist for Bibliometric Analysis 12

**Table S3.** Most Prolific Affiliations and Sources 17

**Table S4.** Author Impact 18

**Table S5.** Themes in the Thematic Map 19


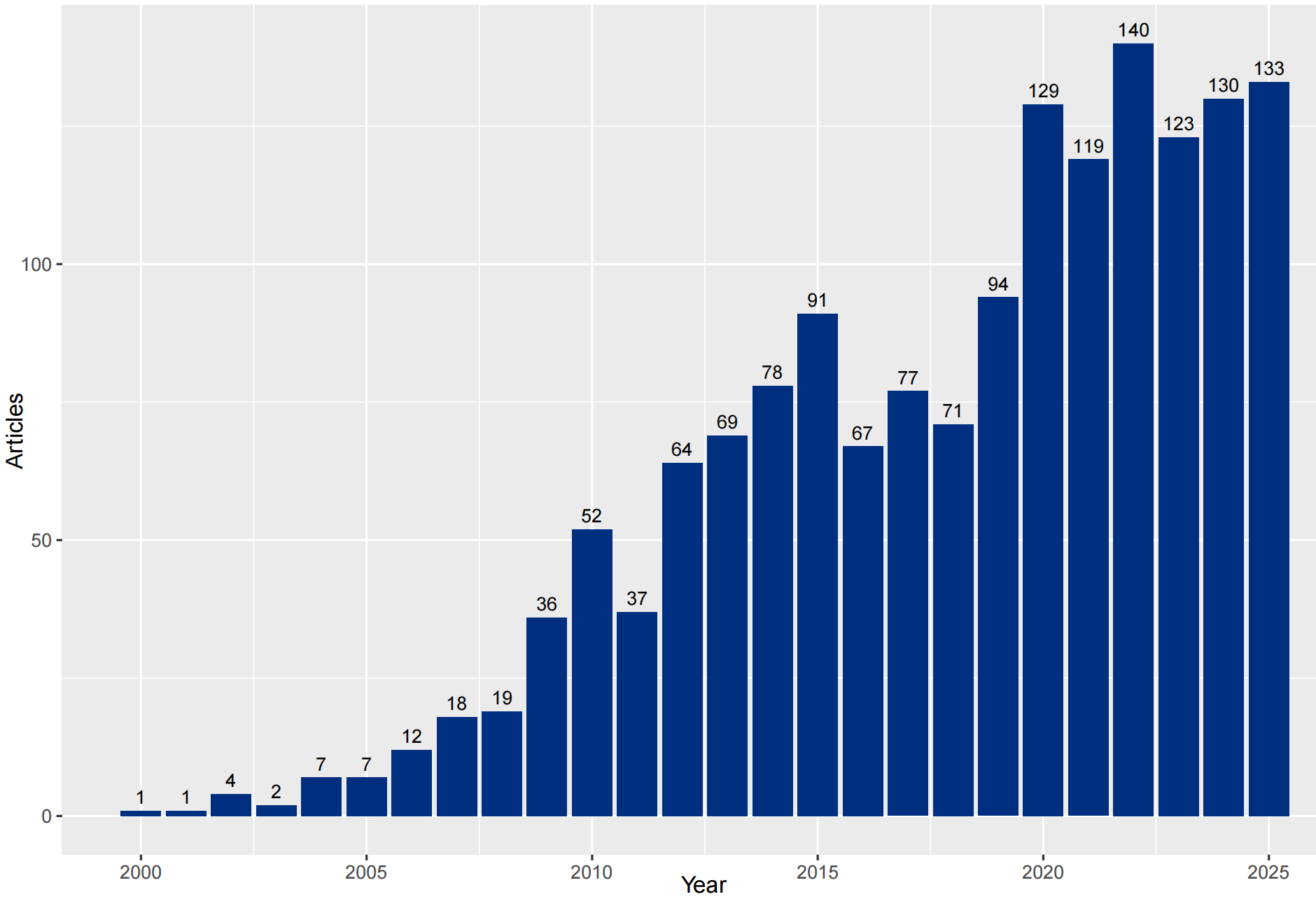


# Figure S1. Annual Publication Volume


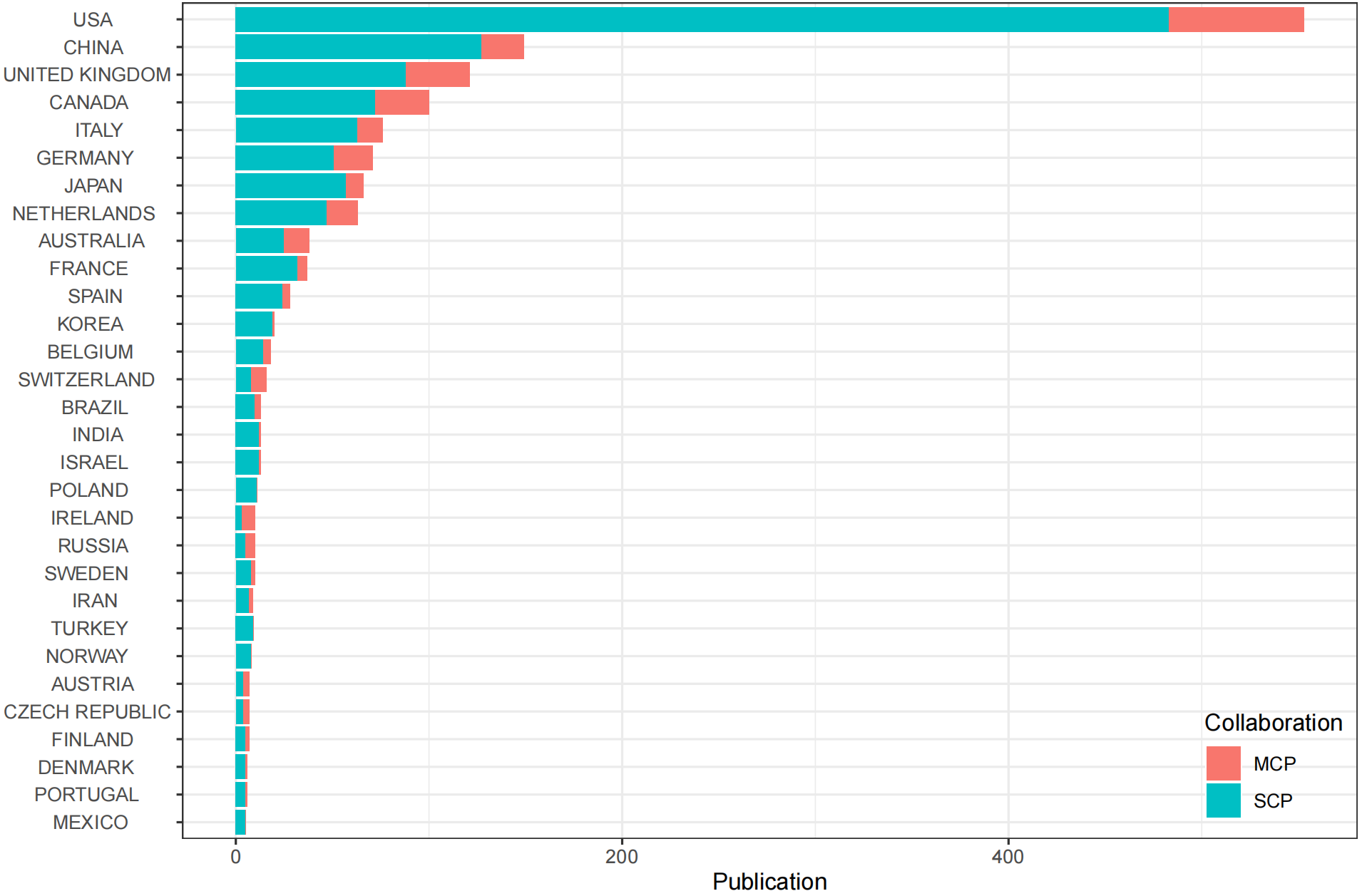


# Figure S2. Bar Chart of Collaboration by Major Countries/Regions

*Note:* The x-axis represents publication output, and the y-axis represents the country/region. The red portion represents Multiple Country Publications (MCP), and the green portion represents Single Country Publications (SCP).


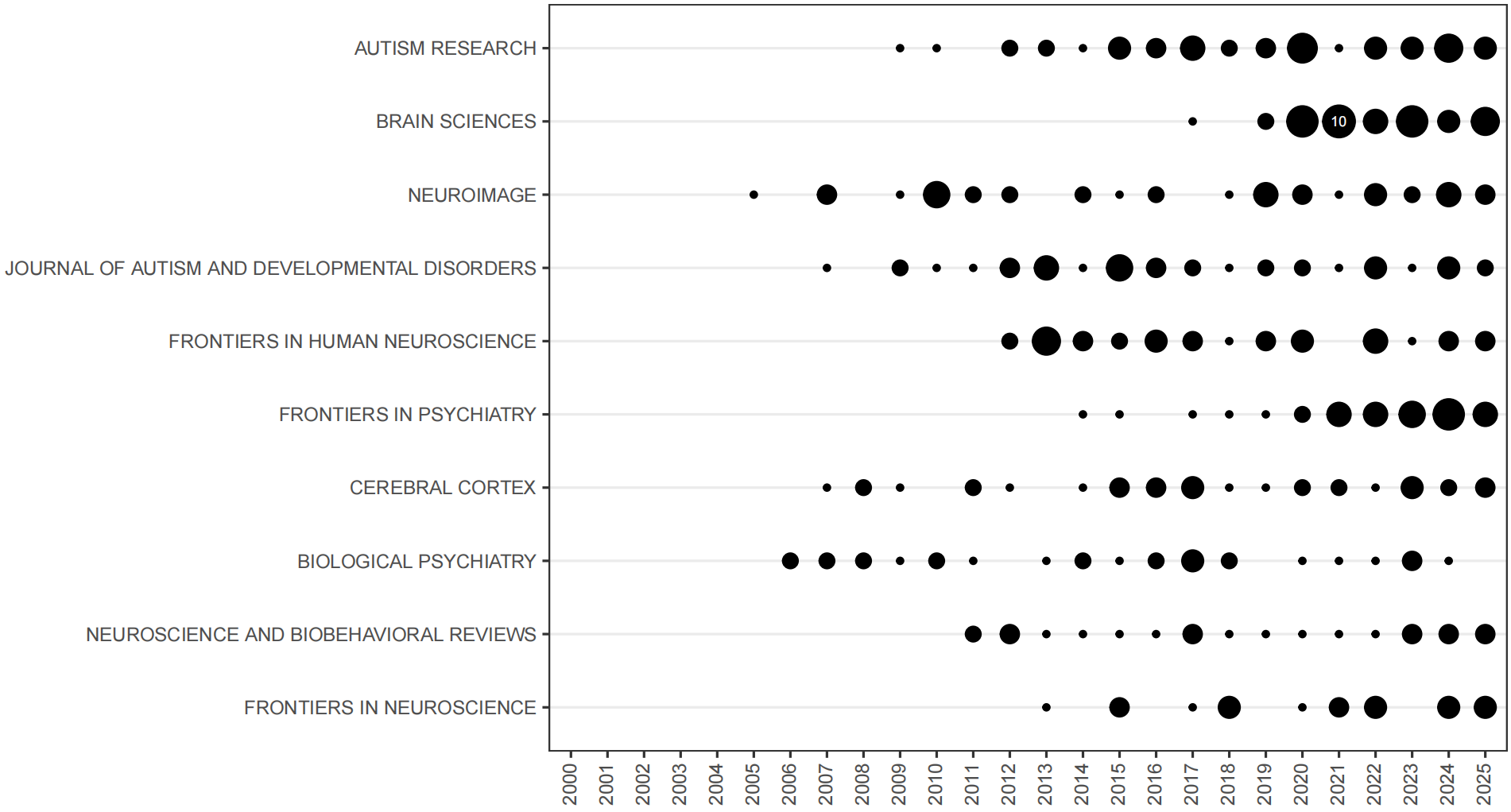


# Figure S3. Dynamic Publication Volume of the Top 10 Journals


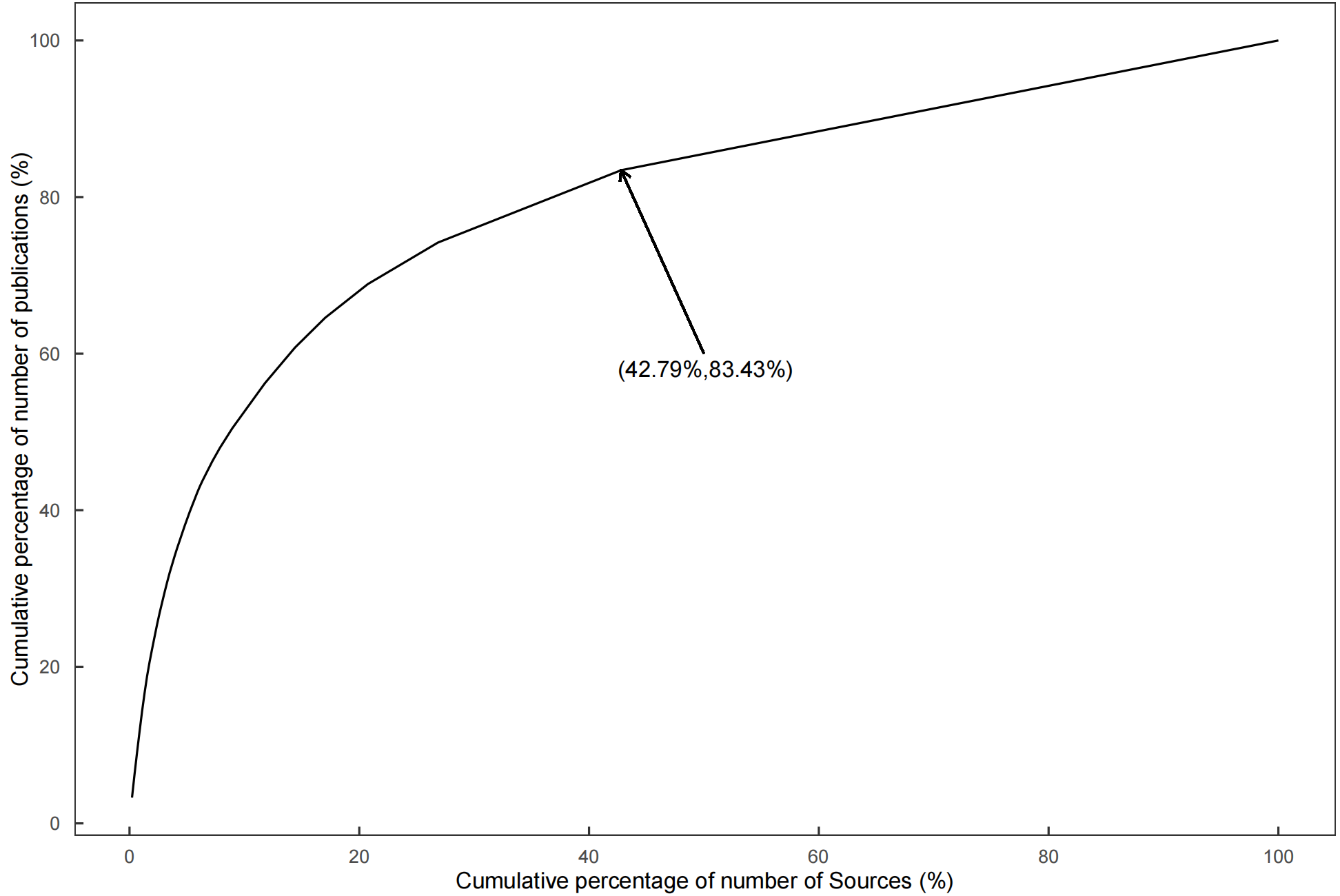


# Figure S4. The Relationship Between Publishing Sources and the Number of Published Articles


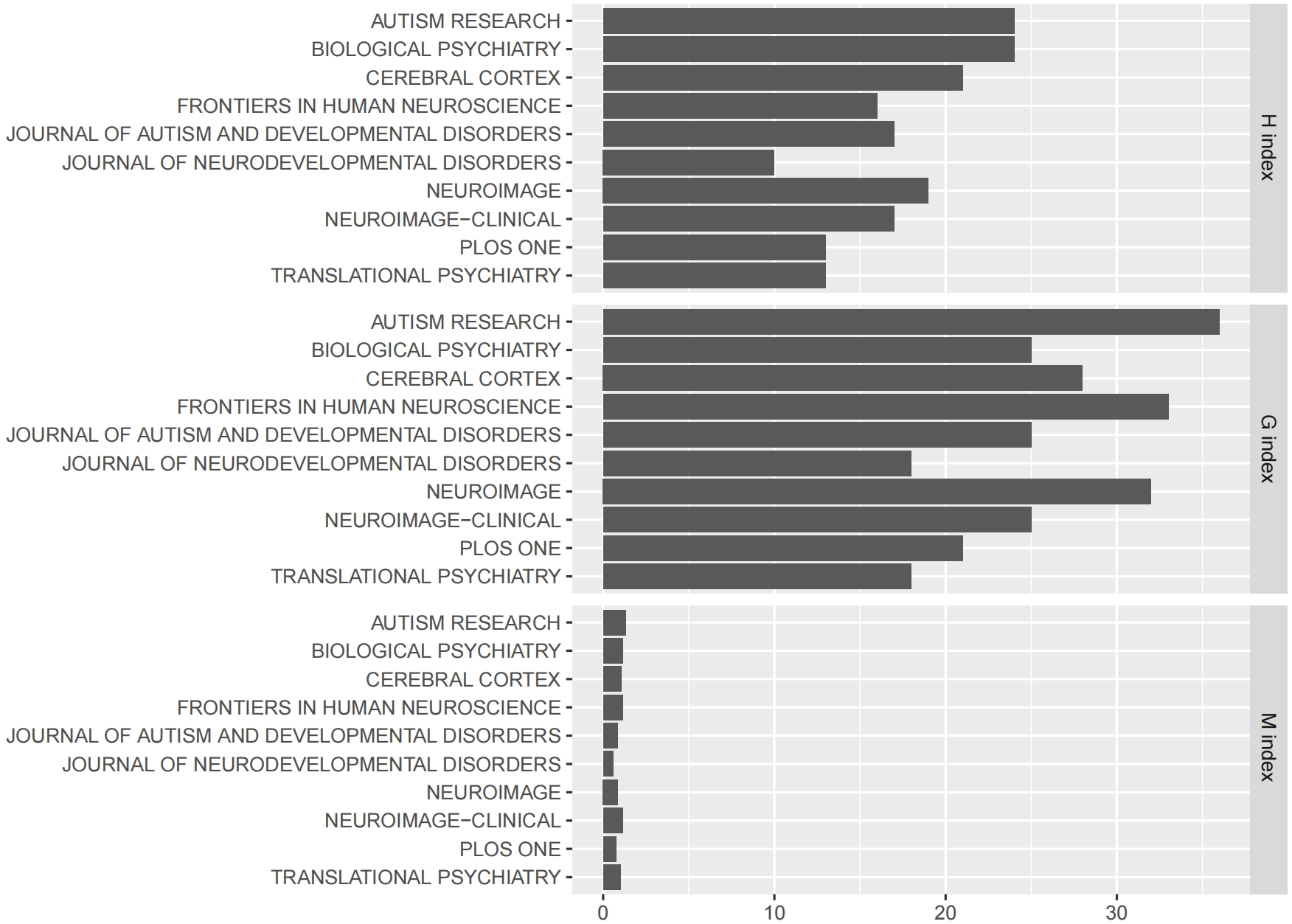


# Figure S5. H-Index and Its Derived Indices (G-Index, M-Index) of Publishing Sources


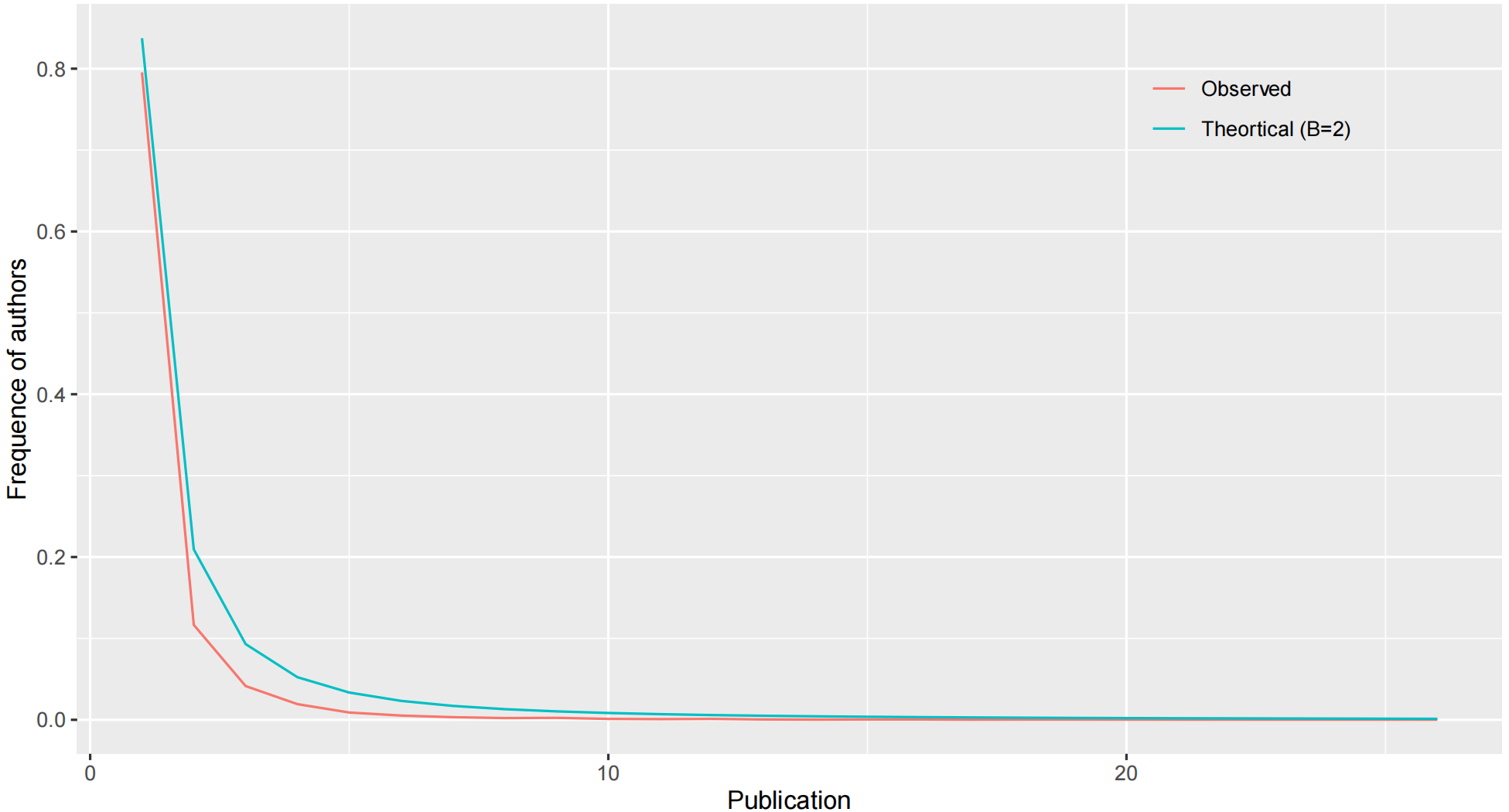


# Figure S6. Lotka's Law Analysis of Publication Distribution


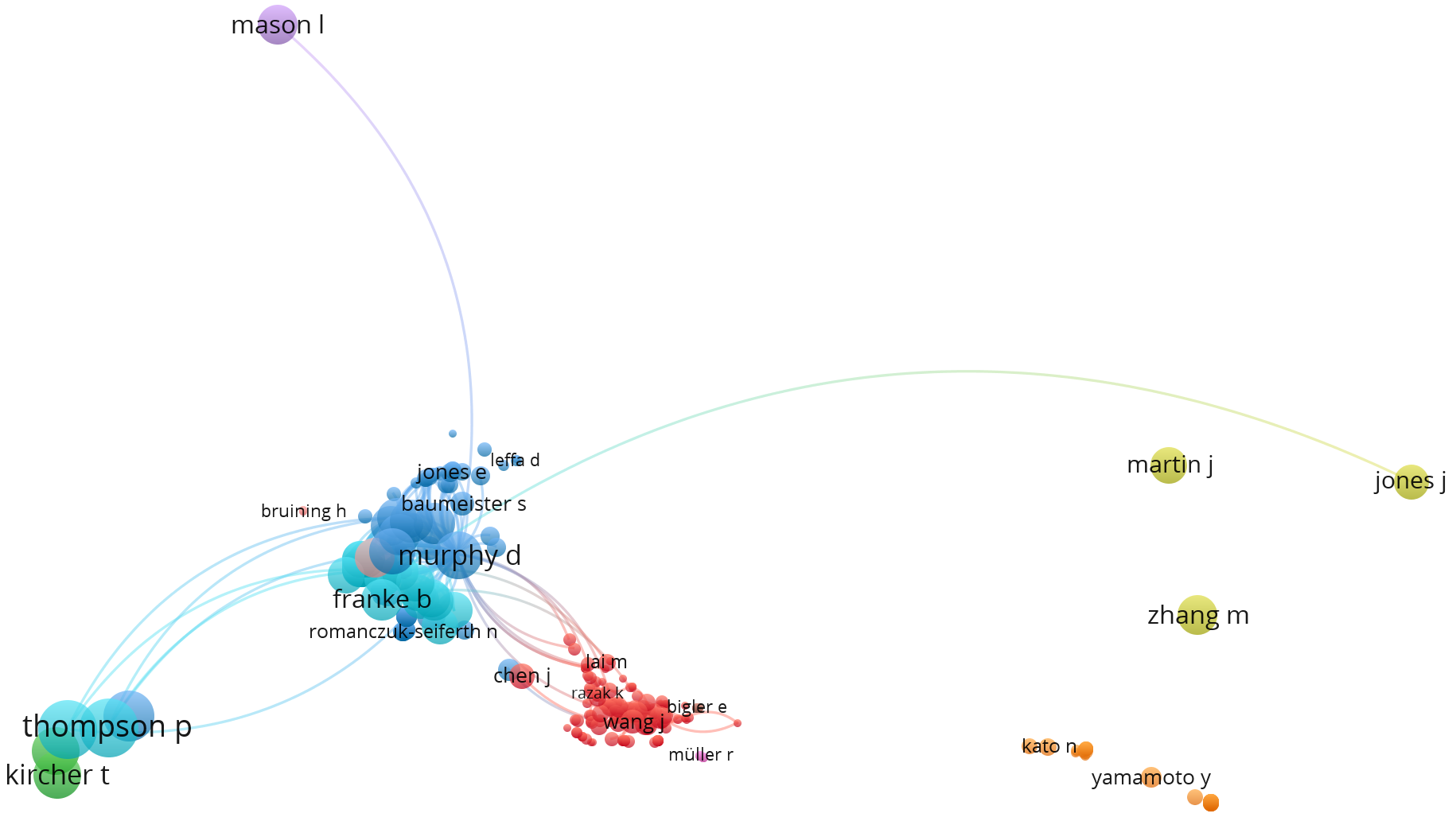


# Figure S7. Author Co-citation Network

*Note:* The color and size of the nodes represent the importance of the authors and the research groups to which they belong, while the connecting lines represent co-citation relationships.


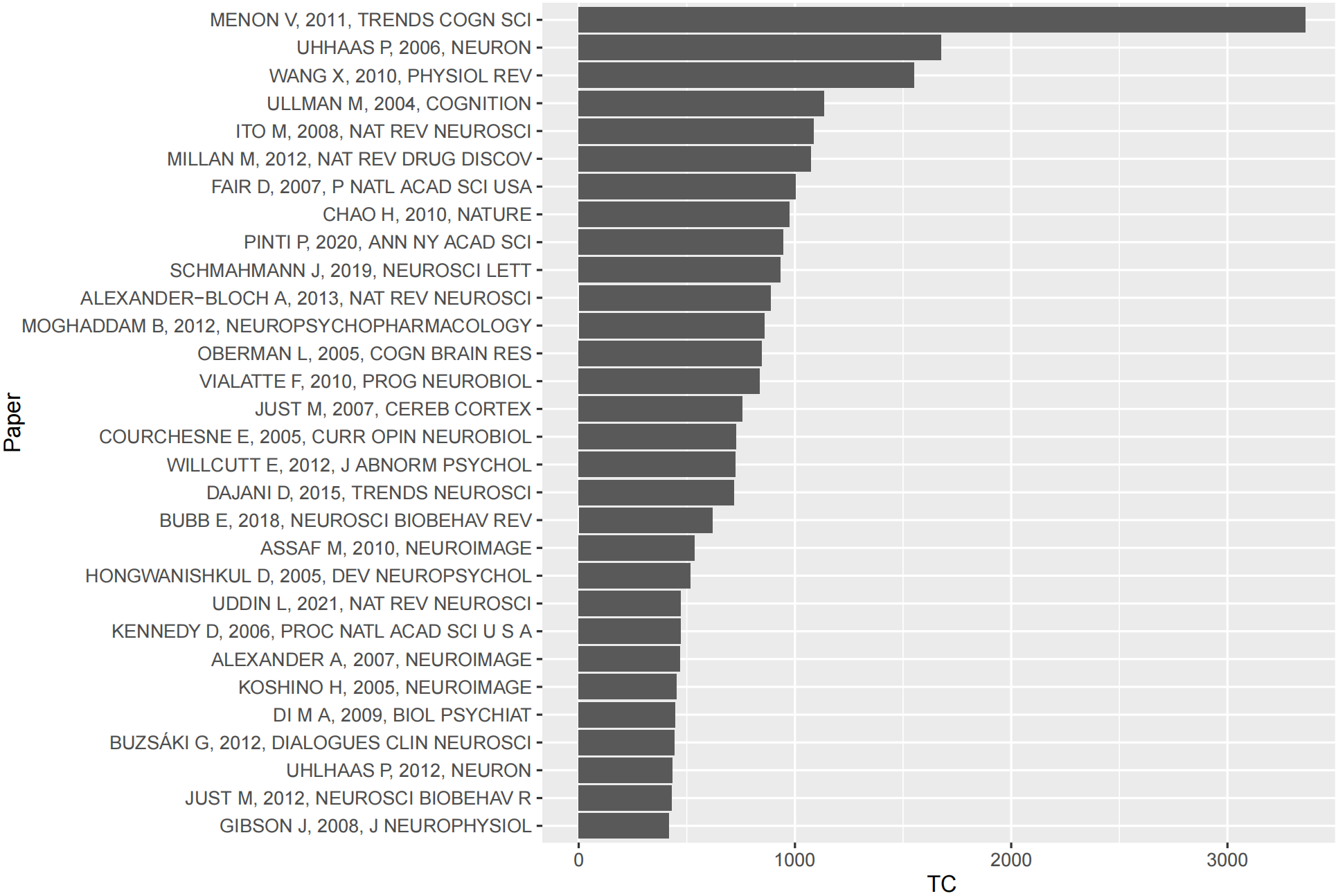


# Figure S8. Highly Cited Documents


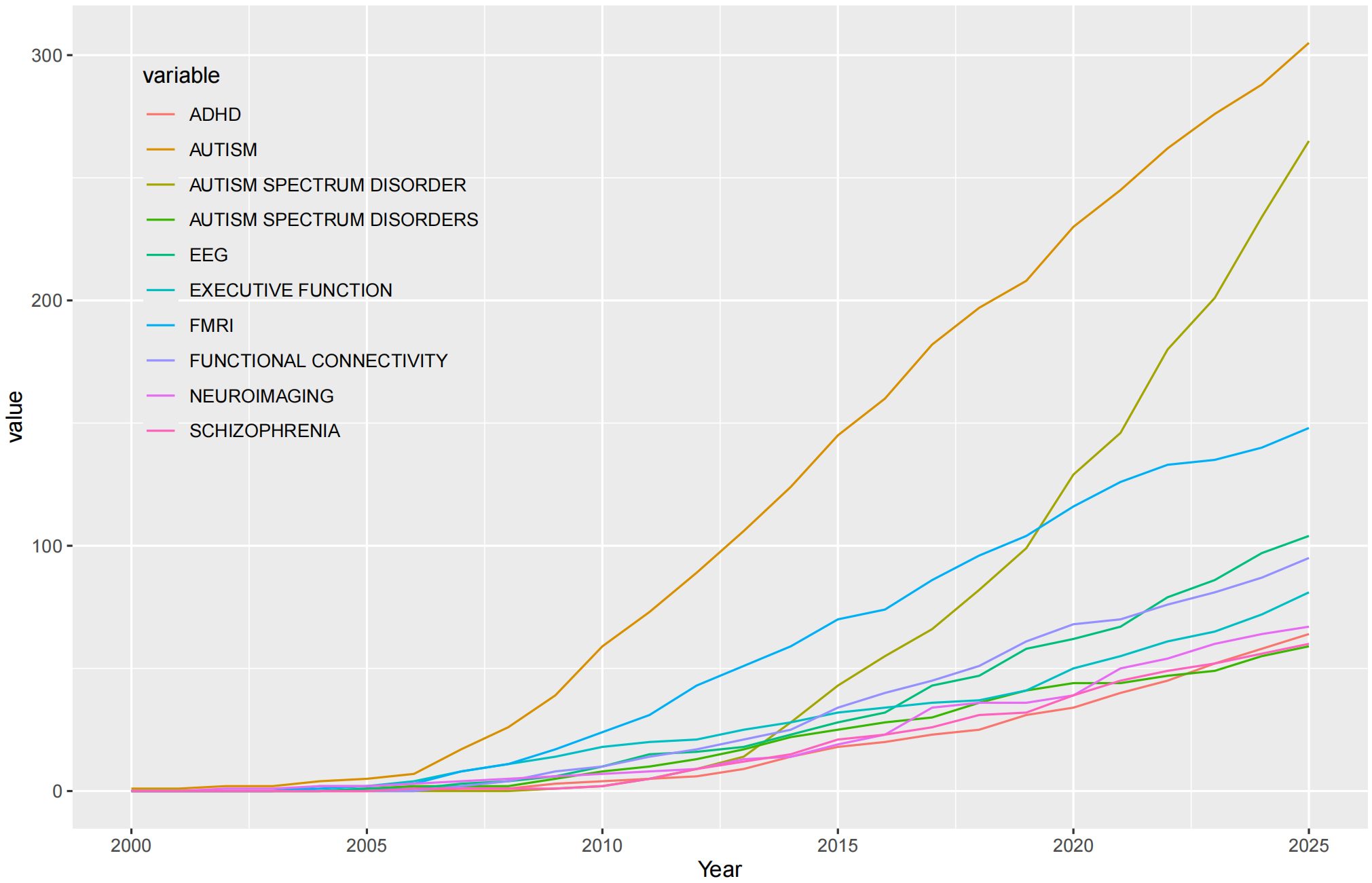


# Figure S9. Keyword Frequency Over Time

# Table S1. Detailed Search Queries for Web of Science and Scopus

| **Database** | **Search Syntax / Query String** |
| --- | --- |
| Web of Science (WoS) | TS = (("autism" OR "autism spectrum disorder" OR "autistic spectrum disorder" OR "ASD" OR "autistic disorder" OR "Asperger syndrome" OR "pervasive developmental disorder") AND ("cognitive control" OR "executive function" OR "executive functioning" OR "working memory" OR "inhibitory control" OR "inhibition" OR "cognitive flexibility" OR "set shifting" OR "cognitive shifting" OR "attention control") AND ("neuroimaging" OR "brain imaging" OR "functional magnetic resonance imaging" OR "fMRI" OR "structural MRI" OR "diffusion tensor imaging" OR "DTI" OR "positron emission tomography" OR "PET" OR "magnetoencephalography" OR "MEG" OR "electroencephalography" OR "EEG")) AND LA=(English) AND DT=(Article OR Review) |
| Scopus | TITLE-ABS-KEY (("autism" OR "autism spectrum disorder" OR "autistic spectrum disorder" OR "ASD" OR "autistic disorder" OR "Asperger syndrome" OR "pervasive developmental disorder") AND ("cognitive control" OR "executive function" OR "executive functioning" OR "working memory" OR "inhibitory control" OR "inhibition" OR "cognitive flexibility" OR "set shifting" OR "cognitive shifting" OR "attention control") AND ("neuroimaging" OR "brain imaging" OR "functional magnetic resonance imaging" OR "fMRI" OR "structural MRI" OR "diffusion tensor imaging" OR "DTI" OR "positron emission tomography" OR "PET" OR "magnetoencephalography" OR "MEG" OR "electroencephalography" OR "EEG"))  AND (LIMIT-TO (DOCTYPE, "ar") OR LIMIT-TO (DOCTYPE, "re"))  AND LIMIT-TO (LANGUAGE, "English") |

# Table S2. PRISMA 2020 Adaptation Checklist for Bibliometric Analysis

| **Section / Topic** | **Item #** | **PRISMA 2020 Item Description** | **Status in this Study** | **Location in Manuscript** |
| --- | --- | --- | --- | --- |
| TITLE | |  |  |  |
| Title | 1 | Identify the report as a systematic review. | Adapted | Title (Identified as "Bibliometric Analysis") |
| ABSTRACT | |  |  |  |
| Abstract | 2 | See the PRISMA 2020 for Abstracts checklist. | Included | Abstract |
| INTRODUCTION | |  |  |  |
| Rationale | 3 | Describe the rationale for the review in the context of what is already known. | Included | Section 1 (Introduction) |
| Objectives | 4 | Provide an explicit statement of the objective(s) or question(s) the review addresses. | Included | Section 1 (Introduction) |
| METHODS | |  |  |  |
| Eligibility criteria | 5 | Specify the inclusion and exclusion criteria for the review and how studies were grouped for the syntheses. | Included | Section 2.1 & 2.2 |
| Information sources | 6 | Specify all databases, registers, websites, organizations, reference lists, and other sources searched or consulted to identify studies. | Included | Section 2.1 |
| Search strategy | 7 | Present the full search strategies for all databases, registers, and websites, including any filters and limits used. | Included | Section 2.1 & Table S1 |
| Selection process | 8 | Specify the methods used to decide whether a study met the inclusion criteria of the review, including how many reviewers screened each record and each report retrieved, whether they worked independently, and if applicable, details of automation tools used in the process. | Included | Section 2.2 (Includes reliability check details) |
| Data collection process | 9 | Specify the methods used to collect data from reports, including how many reviewers collected data from each report. | Adapted | Section 2.4 (Automated metadata extraction via VOSviewer/Bibliometrix) |
| Data items | 10 | List and define all outcomes for which data were sought. | Adapted | Section 2.4 (Bibliometric indicators: citations, keywords, etc.) |
| Study risk of bias assessment | 11 | Specify the methods used to assess risk of bias in the included studies. | N/A | Section 2.3 (Not applicable for bibliometric metadata analysis) |
| Effect measures | 12 | Specify for each outcome the effect measure(s) (e.g. risk ratio, mean difference) used in the synthesis or presentation of results. | Adapted | Section 2.4 (Measures: Citation counts, H-index, Link strength) |
| Synthesis methods | 13 | Describe the processes used to decide which studies were eligible for each synthesis. | Adapted | Section 2.4 (Network analysis & visualization methods) |
| Reporting bias assessment | 14 | Describe any methods used to assess risk of bias due to missing results in a synthesis (arising from reporting biases). | Adapted | Section 4.5 (Discussion of database indexing bias) |
| Certainty assessment | 15 | Describe any methods used to assess certainty (or confidence) in the body of evidence for an outcome. | N/A | Not applicable for bibliometric mapping |
| RESULTS | |  |  |  |
| Study selection | 16 | Describe the results of the search and selection process, from the number of records identified in the search to the number of studies included in the review. | Included | Section 3.1 & Figure 1 |
| Study characteristics | 17 | Cite studies and present characteristics. | Included | Section 3.1 & Table 1 |
| Risk of bias in studies | 18 | Present assessments of risk of bias for each included study. | N/A | Not applicable |
| Results of individual studies | 19 | For all outcomes, present specific results for each study. | Adapted | Section 3.3 (Top cited documents & authors) |
| Results of syntheses | 20 | For all outcomes, present the results of each synthesis. | Included | Section 3 (Bibliometric maps & trends) |
| Reporting biases | 21 | Present assessments of risk of bias due to missing results. | Adapted | Section 4.5 (Limitations regarding database coverage) |
| Certainty of evidence | 22 | Present assessments of certainty (or confidence) in the body of evidence for each outcome. | N/A | Not applicable |
| DISCUSSION |  |  |  |  |
| Discussion | 23a | Provide a general interpretation of the results in the context of other evidence. | Included | Section 4 (Discussion) |
| Limitations of evidence | 23b | Discuss any limitations of the evidence included in the review. | Included | Section 4.5 (Limitations) |
| Limitations of review processes | 23c | Discuss any limitations of the review processes used. | Included | Section 4.5 (Limitations) |
| Implications | 23d | Discuss implications of the results for practice, policy, and future research. | Included | Section 4.4 (Future Outlook) |
| OTHER INFORMATION | | |  |  |
| Registration and protocol | 24 | Provide registration information for the review, including register name and registration number, or state that the review was not registered. | N/A | Not registered (Standard for bibliometric studies) |
| Support | 25 | Describe sources of financial or non-financial support for the review. | Included | Funding Section |
| Competing interests | 26 | Declare any competing interests of review authors. | Included | Conflict of Interest Section |
| Availability of data, code, and other materials | 27 | Report which of the following are publicly available and where they can be found: template data collection forms; data extracted from included studies; data used for all analyses; analytic code; any other materials used in the review. | Included | Data Availability Statement (if applicable in submission system) |

*Note:* This checklist is adapted from the PRISMA 2020 statement to reflect the specific methodology of a bibliometric analysis. Items related to the qualitative synthesis of clinical outcomes (e.g., risk of bias in individual studies, certainty of evidence) are marked as "Not Applicable" (N/A) or "Adapted" where appropriate.

# Table S3. Most Prolific Affiliations and Sources

| **Affiliation** | **N** | **Journals** | **N** |
| --- | --- | --- | --- |
| University of Toronto | 206 | Autism Research | 52 |
| University of California System | 160 | Brain Sciences | 47 |
| University of London | 155 | Neuroimage | 44 |
| Harvard University | 136 | Journal of Autism and Developmental Disorders | 42 |
| King's College London | 136 | Frontiers in Human Neuroscience | 41 |
| Harvard Medical School | 99 | Frontiers in Psychiatry | 37 |
| Harvard University Medical Affiliates | 91 | Cerebral Cortex | 34 |
| University of Pennsylvania | 77 | Biological Psychiatry | 29 |
| University of Cambridge | 61 | Neuroscience and Biobehavioral Reviews | 26 |
| Hospital for Sick Children (Sickkids) | 59 | Frontiers in Neuroscience | 25 |

# Table S4. Author Impact

| **Authors** | **H index** | **G index** | **M index** | **TC** | **NP** | **PY start** |
| --- | --- | --- | --- | --- | --- | --- |
| Murphy D | 20 | 26 | 0.8000 | 3313 | 26 | 2002 |
| Taylor M | 16 | 21 | 1.0667 | 670 | 21 | 2012 |
| Kana R | 12 | 19 | 0.6000 | 2787 | 19 | 2007 |
| Uddin L | 16 | 19 | 0.8889 | 2854 | 19 | 2009 |
| Han Y | 11 | 18 | 0.6111 | 358 | 18 | 2009 |
| Rubia K | 15 | 18 | 0.7143 | 1266 | 18 | 2006 |
| Wang Y | 8 | 17 | 0.6154 | 474 | 17 | 2014 |
| Chan A | 13 | 16 | 0.7222 | 531 | 16 | 2009 |
| Minshew N | 16 | 16 | 0.6400 | 3203 | 16 | 2002 |
| Wang J | 11 | 16 | 1.1000 | 561 | 16 | 2017 |

# Table S5. Themes in the Thematic Map

| **Cluster** | **Callon Centrality** | **Callon Density** | **Terms of the themes** | **Classifications of themes** |
| --- | --- | --- | --- | --- |
| Autism | 0.696 | 10.904 | Autism (15.2), EEG (5.2), Children (2.3), Connectivity (1.6), ERP (1.5), Inhibition (1.2), Fragile X Syndrome (1), Inhibitory Control (0.9), Biomarker (0.9), MEG (0.8) | Motor |
| Autism Spectrum Disorder | 0.641 | 9.710 | Autism Spectrum Disorder (14.9), Electroencephalography (2.6), Neurodevelopmental Disorders (1.7), Magnetic Resonance Imaging (1.5), Magnetoencephalography (1.4), Transcranial Magnetic Stimulation (1), Neurofeedback (1), Adolescence (0.9), Obsessive-compulsive Disorder (0.9), Biomarkers (0.8) | Motor & Basic |
| fMRI | 0.497 | 8.966 | fMRI (12.3), ADHD (5.4), ASD (4.1), Cognitive Control (2.3), Emotion (1.6), Meta-analysis (1.2), DTI (1.1), Response Inhibition (0.9), Asperger Syndrome (0.8), Autistic Disorder (0.7) | Basic |
| Functional Connectivity | 0.493 | 10.006 | Functional Connectivity (9.1), Autism Spectrum Disorders (5.6), Diffusion Tensor Imaging (2.8), Functional MRI (2.2), Default Mode Network (2.1), Resting State (1.8), Brain Development (1.2), fNIRS (1.1), Executive Functioning (1), Structural Connectivity (1) | Motor |
| Executive Function | 1.197 | 9.613 | Executive Function (5), Cognition (3.3), Working Memory (3), Attention (2.4), Development (2.2), MRI (1.9), Executive Functions (1.7), Prefrontal Cortex (1.6), Cognitive Flexibility (1.5), White Matter (1.4) | Basic |
| Neuroimaging | 0.184 | 8.257 | Neuroimaging (15.1), Genetics (3.4), Voxel-based Morphometry (2.7), Cognitive Functions (1.6) | Emerging or Declining |
| Schizophrenia | 0.269 | 11.957 | Schizophrenia (9.8), Dopamine (2), Depression (1.8), Psychiatry (1.7), Oxytocin (1.7), Bipolar Disorder (1.6), Anxiety (1.3), Asymmetry (1.2), Graph Theory (1.1) | Motor |
| Functional Magnetic Resonance Imaging | 0.342 | 8.299 | Functional Magnetic Resonance Imaging (5.5), Language (4.8), Cerebellum (4.7), Neurodevelopment (3.4), Attention Deficit Hyperactivity Disorder (2.2), Tractography (1.2), Event-related Potential (1.1), Dyslexia (1) | Basic |
| Epilepsy | 0.128 | 6.952 | Epilepsy (8.1), Intellectual Disability (3.7), Coherence (2.3) | Emerging or Declining |
| Social Cognition | 0.245 | 10.495 | Social Cognition (5.5), Theory of Mind (3.9), Anterior Cingulate Cortex (2), Empathy (2), Functional Magnetic Resonance Imaging (fMRI) (1.9), Autistic Traits (1.8), Imitation (1.5), Amygdala (1.4), Medial Prefrontal Cortex (1.3), Neuropsychiatry (1.2) | Niche & Motor |
| Autism Spectrum Disorder (ASD) | 0.133 | 8.333 | Autism Spectrum Disorder (ASD) (9.2), Magnetoencephalography (MEG) (2.7) | Emerging or Declining |
| GABA | 0.157 | 6.456 | GABA (11.8), Glutamate (6.3) | Emerging or Declining |
| Comorbidity | 0.055 | 14.011 | Comorbidity (5.4), Neurodevelopmental Disorder (3.4), Treatment (2.4), Diagnosis (2.4) | Niche |
| Adolescents | 0.032 | 10.577 | Adolescents (8.2), Adults (5) | Niche |
| Neuroplasticity | 0.018 | 12.500 | Neuroplasticity (12.2) | Niche |
